# Multiplexed Transcriptomics for Screening Drug Combinations and Defining the Mechanism of Action of HCC Therapeutics at Single‐Cell Resolution

**DOI:** 10.1111/cpr.70148

**Published:** 2025-11-27

**Authors:** Mengmeng Jiang, Haide Chen, Guoxia Wen, Yuqing Mei, Wenzhao Zhou, Bin Xu, Tingyue Zhang, Guangyan Li, Junqing Wu, Xiaoping Han, Xudong Fu, Guoji Guo, Jingjing Wang

**Affiliations:** ^1^ Liangzhu Laboratory Zhejiang University Hangzhou China; ^2^ Bone Marrow Transplantation Center of the First Affiliated Hospital, and Center for Stem Cell and Regenerative Medicine Zhejiang University School of Medicine Hangzhou China; ^3^ Institute of Hematology Zhejiang University Hangzhou China; ^4^ First Affiliated Hospital Zhejiang University School of Medicine Hangzhou China

**Keywords:** anticancer therapy, drug combination, drug screening, HCC, single cell

## Abstract

Compared to classical drug screening, single‐cell screening not only significantly enhances throughput but also provides richer transcriptional response information. In this study, we employed the high‐throughput and high‐sensitive single‐nucleus sequencing platform, snHH‐seq, to screen clinical drug combinations with anti‐hepatocellular carcinoma (HCC) activity. Single‐cell transcriptomics analysis revealed that the HY combination (HHT and YM155) exhibited the strongest suppression of tumour cell proliferation, a finding validated by both in vitro and in vivo functional assays. Further investigation suggested that HY triggers ferroptosis, as evidenced by rescue from cell death upon co‐treatment with the ferroptosis inhibitor Fer‐1. Subcluster analysis identified distinct tumour cell subclusters' responses to HY treatment. A gene regulatory network analysis highlighted JUN as a key regulator mediating proliferation inhibition, primarily active in the apoptotic cell subcluster. These findings illustrate how integrating high‐throughput screening with mechanistic dissection can accelerate the discovery of targeted drug combination therapies, and offer a blueprint for precise interventions using pathway vulnerabilities and cellular heterogeneity in HCC.

## Graphical abstract

We designed a framework for screening clinical drug combinations with anti‐hepatocellular carcinoma (HCC) activity, comprising four parts: primary screening, single‐cell screening, functional validation, and mechanism research. High‐throughput single‐cell screening identifies HY (HHT and YM155) as a potent anti‐HCC drug combination, validated by in vitro and in vivo functional assays.

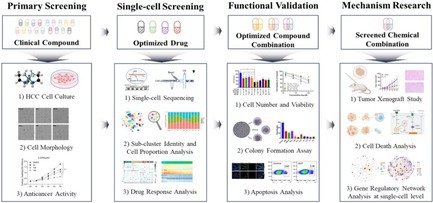

## 1 Introduction

The advent of single‐cell sequencing technology has revolutionised life sciences research, much like the leap from conventional microscopy to super‐resolution imaging enabled unprecedented precision in visualising biological structures [1, 2]. This technology now empowers researchers to address fundamental questions with single‐cell resolution, unlocking a very broad range of applications such as construction of cell atlases for various species, analysis of cellular heterogeneity within tissues, discovery of novel cell subpopulations, identification of disease biomarkers, and reshaping of patient stratification [3, 4, 5, 6, 7, 8, 9, 10].

In the field of high‐throughput drug screening, single‐cell sequencing technology offers distinct advantages over traditional bulk transcriptomic or phenotypic approaches. One key advantage is high‐throughput detection, allowing a single experiment to test multiple drugs across different doses, time points, concentrations, and combinations. Moreover, it captures rich transcriptional responses information, revealing drug‐targeted subpopulations, cluster‐specific transcriptional changes, pathway perturbations, and dose–response spectra. Critically, this approach eliminates batch effects by reducing operational errors between experiments and reduces costs by consolidating screening into a unified workflow. For instance, McFarland et al. [11] leveraged single‐cell RNA sequencing technology (scRNA‐seq) to achieve high‐throughput, multi‐dimensional analysis of cancer cells' transcriptional responses to drug perturbations. Srivatsan et al. [12] treated three tumour cell lines with 188 different drugs, capturing approximately 650,000 single‐cell transcriptomes in a single experiment, systematically revealing the mechanisms of action for multiple types of drugs using sci‐Plex. Furthermore, single‐cell sequencing technology is widely applied in the mechanistic elucidation of precision medicine for disease treatment. In cervical cancer research, scientists delineated eight cell types and five subpopulations of malignant epithelial cells, validated PLOD2 as a prognostic gene with therapeutic potential [13]. In the study of muscle regeneration, Feng et al. [14] revealed that Zn‐DHM treatment increased the expression of M2 macrophage markers and enhanced the proliferation and differentiation capacity of muscle stem cells by single‐cell profiling [14].

Globally, hepatocellular carcinoma (HCC) is one of the most malignant tumours with high incidence and mortality rates, posing a significant threat to human health [15, 16, 17]. Due to its covert progression and rapid development, most patients are diagnosed at end‐stage, resulting in low surgical resection rates, limited therapeutic efficacy, and poor prognosis. Empirical evidence has shown that combination targeted therapies have emerged as a promising strategy to address these challenges [18, 19]. A study involving 47 end‐stage HCC patients demonstrated that the combination of sintilimab (anti‐PD‐1 antibody) and apatinib (VEGFR2 inhibitor) as a first‐line treatment achieved good anti‐tumour efficacy and safety [20]. Similarly, research on 63 HCC patients showed that the combination of tyrosine kinase inhibitors (TKIs) and anti‐PD‐1 antibodies significantly improved clinical outcomes for most patients [21, 22]. Additionally, clinical drugs originally used for haematological malignancies are also being expanded to the treatment of liver cancer [23]. For instance, arsenic trioxide is the first‐line therapy for acute promyelocytic leukaemia and is approved for advanced primary liver cancer [24]. Mitoxantrone, which was originally used to treat leukaemia, lymphoma, and other hematologic cancers, has expanded its indications to include solid tumours such as liver cancer and bladder carcinoma [25]. However, these drugs have notable limitations. For example, arsenic trioxide may elevate transaminase levels during liver cancer treatment, necessitating the concurrent use of liver‐protective agents and close monitoring of liver function. Such side effects, combined with the potential emergence of drug resistance, restrict their clinical application [26]. There is an urgent need for novel drugs or optimised combination therapies to overcome these limitations and expand treatment accessibility. In this context, single‐cell sequencing technology holds promise for expediting the development and optimised combination of drugs for HCC, addressing drug screening challenges by identifying effective drug combinations and understanding the mechanisms of drug resistance at single‐cell resolution.

We have developed a high‐throughput and highly sensitive single‐nucleus RNA sequencing method called snHH‐seq [27]. This approach integrates random primers and a pre‐indexing strategy on a droplet microfluidic platform, enabling total RNA detection in single nuclei from clinically frozen samples. Species‐mixing experiments demonstrated that snHH‐seq produces high‐fidelity single‐cell libraries with a doublet rate no higher than 0.8%. Unlike poly(A)‐based 10X Chromium, snHH‐seq achieves uniform coverage across gene bodies and allows tracing of mutations at single‐cell resolution in clinical specimens. When applied to tumour samples, snHH‐seq effectively depletes cytoplasmic rRNA without additional removal steps and efficiently captures nascent RNA with intron retention. Compared to Microwell‐seq, snHH‐seq detects a higher proportion of protein‐coding transcripts, transcription factors, lncRNAs, non‐polyadenylated genes, and sncRNAs, while nuclear isolation yields lower cellular stress signals and longer transcript capture.

In this study, we utilized snHH‐seq, a high‐throughput and high‐sensitivity single‐cell sequencing platform, to systematically screen clinical compounds for their anti‐HCC activity. We uncovered a novel combination therapy regimen—HHT and YM155 (designated HY)—that synergistically exerted the most potent inhibitory effect on cellular proliferation. The potent anti‐tumor efficacy of HY was validated through comprehensive in vitro and in vivo functional experiments. Importantly, single‐cell resolution analysis revealed the heterogeneous transcriptional response to HY treatment across HCC cellular subpopulations and elucidated its underlying molecular mechanisms. Together, this study not only establishes HY as a promising novel combination therapy regimen for HCC but also provides mechanistic insights at the single‐cell level, advancing our understanding of precision oncology approaches for liver cancer treatment.

## 2 Materials and Methods

### 2.1 Cell Culture and Reagents

Human hepatocellular carcinoma HepG2 cells were sourced from the Zhijiang Laboratory (Hangzhou, China) without mycoplasma contamination. The HepG2 cells were cultured in DMEM with 10% FBS and 1% Penicillin and Streptomycin solution. Cells were incubated in a humidified atmosphere incubator of 5% CO_2_ at 37°C. Cladribine (T2558), homoharringtonine (T3380), azacitidine (T1339), cytarabine (T1272), venetoclax (T2119), sepantronium bromide (T2111), selinexor (T6106), ruxolitinib (T1829), panobinostat (T2383), idarubicin hydrochloride (T6010), daunorubicin (T1511L), ferrostatin‐1 (T6500), necrostatin‐1 (T1847), Z‐VAD(OH)‐FMK (T7020), and T‐5224 (T5416) were purchased from TargetMol (Boston, United States).

### 2.2 Cell Growth Inhibition Assay

Cells (5 × 10^3^ cells/well) were seeded in 96‐well plates, cultured overnight to allow cell attachment, and treated with different drug compounds. After 48 h of treatment, 10 μL of CCK‐8 dye was added into each well to a final concentration (v/v) of 10% and incubated for another 2 h. Subsequently, the absorbance (OD) was measured at 450 nm by the SPARK microplate reader of the multi‐wavelength measurement system (TECAN, Switzerland). Cell growth inhibition rate was evaluated as the ratio of the absorbance of the treated samples to that of the negative control samples and analyzed by GraphPad Prism 8.0.2 software. All experiments were carried out in triplicate.

Dose–response curves and IC50 calculations were also performed using GraphPad Prism 8.0.2 software. The methodology involved the following steps: Cell viability values at multiple specific concentrations were input into the software. The X‐axis was converted to a logarithmic scale. By selecting Analyse > Nonlinear Regression (curve fit) from the toolbar, the [Inhibitor] versus normalized response under the Model of Dose–Response‐Inhibition category was applied for data analysis.

### 2.3 Colony Forming Assay

Cells were seeded in 6‐well plates at 1 × 10^5^ cells/well and incubated overnight. The cells were next treated with different drug compounds. The culture medium was changed every 3–4 days until there were colonies visible to the naked eye (about 14 days). After that, the culture solution was discarded, 4% paraformaldehyde was added to the wells and the cells were fixed for 10 min. After fixation, the cells were washed with distilled water and stained with crystal violet staining solution for 10 min. After staining, the staining solution was removed through repeated washing. Photos were taken and the formation of cell clones in each group was compared by using Image J.

### 2.4 Apoptosis Assay

Cells were seeded in a 96‐well clear bottom black plate at a density of 5 × 10^3^ cells/well and cultured overnight. Following attachment, cells were treated with different drug compounds. The control group was treated with complete medium only. Each treatment group had three replicates. After treatment for 48 h, 1 μL Hoechst 33342, 5 μL PI, and 5 μL FITC‐Annexin V were added to each well. Apoptosis in each group was evaluated and analyzed by Operetta CLS High Content Screening reader (PerkinElmer, United Kingdom). The Excitation/Emission wavelengths for Hoechst 33342, FITC‐Annexin V, and PI were 350/461 nm, 494/518 nm, and 535/617 nm, respectively.

### 2.5 FCM Analysis

Cells were seeded in a 6‐well plate at a density of 1 × 10^5^ cells/well and cultured overnight. Following attachment, cells were treated with different drug compounds. The control group was treated with complete medium only. After treatment for 48 h, cells were stained in PBS supplemented with 2% fetal bovine serum (FBS) at 4°C for 30 min with Annexin V‐FITC and PI. Stained cells were washed once with PBS supplemented with 2% FBS and analyzed using the BD LSR Fortessa (New York, United States). The proportion of positive/negative cells with the same mean fluorescence intensity was represented.

### 2.6 Real‐Time Cell Proliferation Analysis

HepG2 cells were seeded in accompanying 96‐well plates at a density of 2 × 10^3^ cells/well, cultured overnight to allow cell attachment, and treated with different drug compounds. The value of the cell proliferation signal was detected by the Intelligent Cell Real‐time Monitoring System (Shanghai, China) according to the User Operation Manual.

### 2.7 Single‐Cell Screening

HepG 2 cells were seeded in a 6‐well plate at a density of 1 × 10^5^ cells/well and cultured overnight. Following attachment, cells were treated with different drug compounds. The control group was treated with complete medium only. After treatment for 48 h, cells were collected and snHH‐seq was performed for single‐cell screening [27]. The general experimental workflow for snHH‐seq is as follows: Isolate cell nuclei, perform counting, and suspend them in a reverse transcription mixture (RT mix). For a 96‐well plate reaction, 110× RT mix was prepared: 55 μL 10 mm dNTP, 484 μL RT buffer, 55 μL RNA Inhibitor (Vazyme), 55 μL Reverse Transcriptase, 341 μL Wash Buffer. The reverse transcription kit was included in the VITAPilote‐EFT1200 kit (Cat # R20122124) ordered from M20 Genomics. Both nuclei‐RT mix (≤ 50,000 nuclei, 9 μL per well) and 10 μm well‐specific barcoded RT primers (1 μL per well) were distributed to each well of the 96‐well plate and stirred gently with the pipette tip. The reaction mix was incubated with the thermal cycling: (8°C for 12 s, 15°C for 45 s, 20°C for 45 s, 30°C for 30 s, 42°C for 2 min) × 10 cycles, 42°C for 45 min. After the reaction, all nuclei were collected, mixed, and washed using PBST (PBS, 0.05% Tween 20) three times to remove the residual primers. After washing, nuclei were suspended in TdT mixture (100,000–1,000,000 nuclei per reaction, 39 μL nuclei in PBST, 5 μL 10 × TdT buffer (NEB), 5 μL CoCl2 (NEB), 0.5 μL 100 mm dATP (Invitrogen), 0.5 μL TdT enzyme (NEB)). The TdT reaction mix was incubated at 37°C for 30 min. After the reaction, nuclei were washed using PBST three times. The nuclei were counted and diluted to 2000–8000 nuclei h−1 using OptiPrep (Stem Cell). DNA extension reaction mixture was prepared (for 80 μL): 40 μL ddH2O, 16 μL thermopol buffer, 6 μL 10 mm dNTP, 6 μL BST 2.0 Warmstart (NEB), 6 μL RnaseH (NEB), 6 μL USER (NEB). Nuclei, 2 × DNA extension reaction mixture (M20 Genomics, VITAPilote‐EFT1200 kit), and barcoded beads were encapsulated into droplets using the microfluidic platform. All the required reagents for the droplet reaction can be ordered from M20 Genomics company. The flow rates: 200 μL h−1 for nuclei/reaction mixture; 500 μL h − 1 for oil; 50 μL h − 1 for beads. The mean value of droplet volume is 0.48 nL. The droplets (20–50 μL per tube) were incubated at 37°C for 1 h, 50°C for 30 min, 60°C for 30 min, 75°C for 20 min. Then the droplets were broken by mixing with equal amounts of 20% PFO (1H,1H,2H,2HPerfluoro‐1‐octanol, Sigma). The supernatant was collected after centrifuging and purified with 1.2 × DNA Clean Beads (Vazyme) and eluted in 40 μL ddH2O. Two rounds of PCR were performed to amplify cDNA and add sequence adapters. The amplified libraries were purified with 0.8 × DNA Clean Beads and quantified using Qubit (Invitrogen). Circularization was performed to obtain a sequencing nanoball library for MGI DNBSEQ using VAHTS Circularization Kit for MGI (Vazyme, NM201). Library sequencing was performed using DNBSEQ‐T7 with paired‐end reads of 100 or 150 bp.

### 2.8 Single‐Cell Data Preprocessing

For sequencing library, the poly‐A tail was trimmed from each raw sequencing read using Cutadapt. Subsequently, real cells were identified based on the number of reads per cell, utilising a manually defined minimal read cutoff determined by the results of the UMI‐tools whitelist function. Reads were then aligned to the GRCh38 reference genome using the STAR 2‐pass mode, and only uniquely mapped reads were retained. Each read was assigned to its corresponding gene using the “gene” tag within the GRCh38 GTF by employing feature Count. The digital gene expression (DGE) was generated using the UMI tools count function. Low‐quality cells were filtered out using thresholds for UMIs (200 < nCount_UMI < 2500) and mitochondrial gene count (percent mt < 50%). Genes detected in fewer than 0.25% of the cells were removed. Following this quality control and data preprocessing, 46,901 high‐quality cells were collected for analysis, with an average UMI count of 681 and an average gene count of 332.

### 2.9 Clustering of Single‐Cell Data Matrix

Seurat [28] was used to perform clustering analysis of single‐cell data. DGE data were used as inputs. Cells from the pre‐processed data and genes expressed in more than three cells were selected for further analysis. Filtered data were ln(CPM/100 + 1) transformed, and the number of UMI and the percentage of mitochondrial gene content were regressed out. The resulting clustering results were visualised using Uniform Manifold Approximation and Projection (UMAP). The default Wilcoxon rank‐sum test was used by running the FindAllMarkers function in Seurat to find differentially expressed markers in each cluster. The heat map produced by the DoHeatmap function is one basis for judging the quality of clustering. The dot plot map produced by the DotPlot function is performed to show the specific gene expression in each subpopulation with different drug treatment.

### 2.10 Regulon Activity Analysis

To determine the ‘on/off’ activity of each regulon in each cell type, we used area under curve (AUC) scores for each regulon as a threshold to binarize the regulon activity scores by SCENIC [29]. The protocol consists of three stages: (1) coexpression modules are inferred using a regression per‐target approach (GRNBoost2); (2) the indirect targets are pruned from these modules using cis‐regulatory motif discovery (cisTarget); (3) the activity of these regulons is quantified via an enrichment score for the regulon's target genes (AUCell). The regulon activity t‐SNE maps were created using the function tsneAUC in the R package SCENIC with the binary regulon activity matrix. To connect regulons with cell types, we used the Wilcoxon rank‐sum test to identify cell‐type‐specific regulons with AUC score matrices.

### 2.11 TF Regulatory Network Inference

An analytical strategy [30] has been developed for inferring TF regulatory networks in single‐cell RNA‐seq, and we applied this method to our snHH‐seq dataset. The goal of this analysis is to identify potential regulatory links between TFs and their target genes based on patterns observed in a single‐cell dataset. This analysis can be broken down into five key steps: (1) compute de‐noised metacell expression profiles from the single‐cell dataset; (2) scan gene promoters for the presence of TF motifs; (3) model gene expression as a function of TF expression; (4) assemble TF regulons and regulatory networks; (5) downstream analysis of TF regulatory networks. Open‐source implementation of this TF regulatory network inference algorithm could be obtained from hdWGCNA R package [31].

### 2.12 Statistical Analysis

**FIGURE 1:**
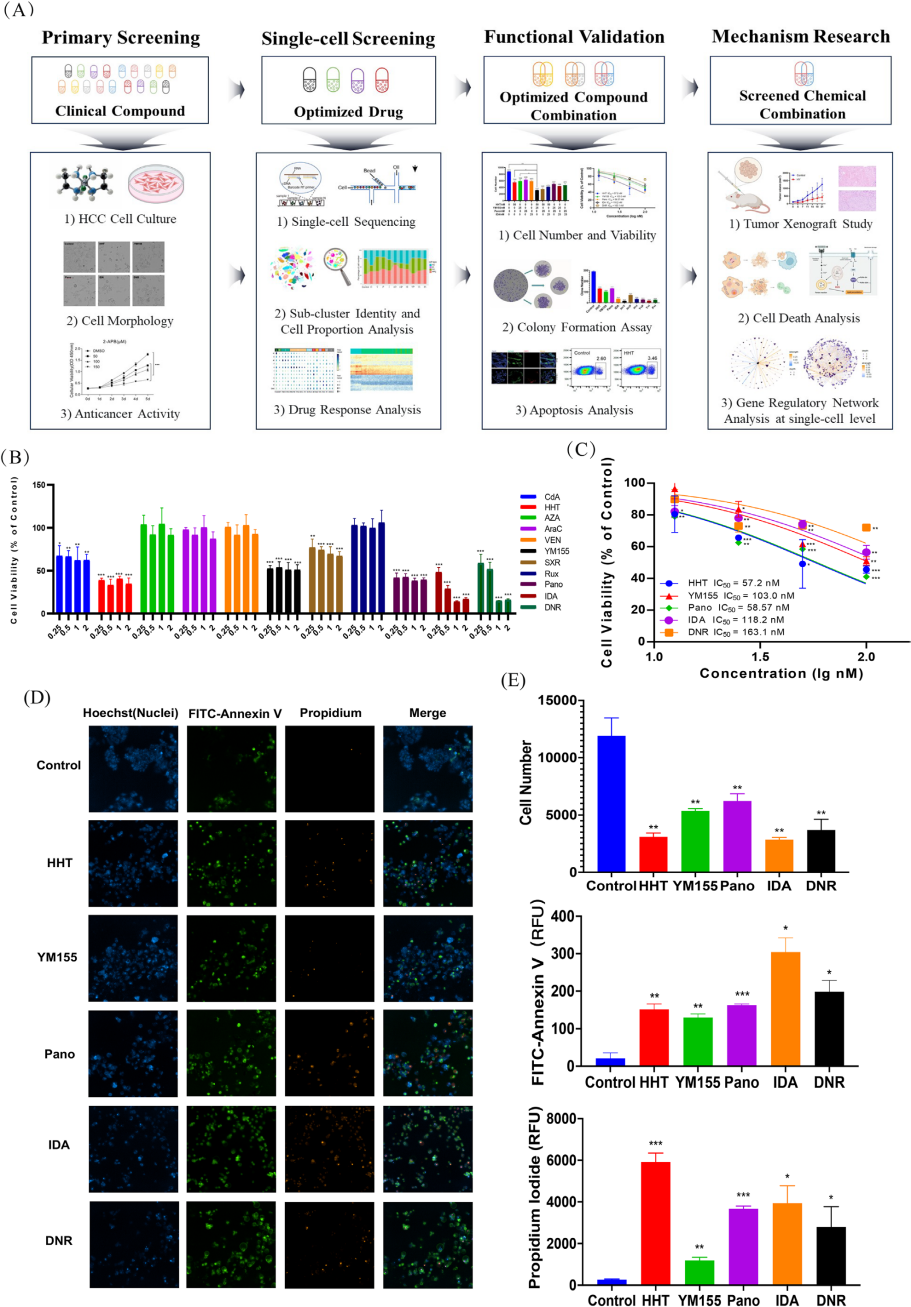
Drug screening identified compounds that effectively inhibit HCC cell proliferation. (A) Design framework diagram of this study, which includes four parts: Primary screening, single‐cell screening, functional validation, and mechanism research. (B) Cell viability percentage (%) of different concentrations of drug against HepG2 cell line. Cells were seeded in a 96‐well plate and treated with 0.25, 0.5, 1, and 2 μM drug for 48 h, and the cell viability percentage was detected with CCK‐8 kit. (C) Cell viability percentage (%) of HHT, YM155, Pano, IDA, and DNR against HepG2 cell line. Cells were seeded in a 96‐well plate and treated with 12.5 nM, 25 nM, 50 nM, and 100 nM drug for 48 h, and the cell viability percentage was detected with CCK‐8 kit. The dose–response curves were performed using GraphPad Prism 8.0.2 software. The half‐maximal inhibitory concentration (IC50) refers to the concentration of a drug that induces 50% apoptosis in cells. (D) Representative images of cell apoptosis in various groups. Different drugs were treated on HepG2 cells for 48 h, and then apoptosis was detected by Hoechst 33342/FITC‐Annexin V/PI triple staining. The images were collected by an Operetta CLS HCS reader. Merged images were the composition of images of the same field from different channels. (E) Cell number, fluorescence intensity of FITC‐Annexin V and PI in HepG2 cells treated by HHT, YM155, Pano, IDA, and DNR. Data are presented as mean ± SD from three independent experiments (*n* = 3). Statistical comparisons are performed using unpaired two‐tailed Student's t‐test. Significance levels are denoted as follows: *p < 0.05, **p < 0.01, ***p < 0.001. The absence of an asterisk (*) indicates no significant difference.

In Figure 1B,C, the statistical significance of hepatocellular carcinoma cell viability following treatment with drug concentrations of 12.5, 25, 50, and 100 nM was evaluated using an unpaired two‐tailed Student's *t*‐test. In Figure 1E, following treatment of HepG2 cells with HHT, YM155, Pano, IDA, and DNR, the two‐tailed Student's *t*‐test was applied to assess differences in cell number, FITC‐Annexin V fluorescence intensity, and PI fluorescence intensity. Data are presented as mean ± SD (*n* = 3). Significance levels are indicated as follows: *p < 0.05, **p < 0.01, ***p < 0.001. In Figure 3A,B, statistical significance for both cell number and growth inhibition was assessed by the two‐tailed Student's *t*‐test. Specific comparisons are indicated as follows: *p < 0.05, **p < 0.01 versus the negative control group; #*p* < 0.05, ##*p* < 0.01 versus the HHT alone group; $*p* < 0.05, $$*p* < 0.01 versus the YM155 alone group; %*p* < 0.05, %%*p* < 0.01 versus the Pano alone group; &*p* < 0.05, &&*p* < 0.01 versus the IDA alone group. In Figure 3E, the statistical significance of clone number was determined by the two‐tailed Student's *t*‐test (*p < 0.05, **p < 0.01, ***p < 0.001). In Figure 3L, the statistical significance of cell viability was also determined by the two‐tailed Student's *t*‐test (*p < 0.05).

## 3 Results

### 3.1 Drug Screening Identified Compounds That Effectively Inhibit HCC Cell Proliferation

The efficacy of drugs used clinically for malignant haematological diseases in treating solid tumours remains an area worthy of further exploration. In this study, we integrated classical high‐content screening platforms with scRNA‐seq technologies to identify drug combinations that effectively inhibit the proliferation of HCC cells (Figure 1A). We first conducted an in vitro inhibition assay on HCC cells using targeted agents that have been clinically applied for the treatment of leukaemia (Table S1). Systematic analysis of clinical compounds acting on HCC cells revealed significant cytostatic effects by CdA, HHT, YM155, SXR, Pano, IDA and DNR, while AZA, AraC, VEN and Rux showed no significant changes in inhibiting HCC cell viability (Figure 1B). The IC50 value serves as a measure of a drug's ability to induce apoptosis—the stronger the induction capability, the lower the numerical value. Then, we adjusted the concentration gradients for subsequent cell proliferation assays. The results indicated that HHT, YM155, Pano, IDA, and DNR exhibited potent anti‐cancer activity at lower concentrations, with IC_50_ values of 57.2, 103.0, 58.57, 118.2, and 163.1 nM, respectively (Figure 1C and Figure S1A). AZA, AraC, VEN, and Rux continued to show no significant changes in inhibiting HCC cell viability even at higher concentrations (Figure S1B).

To further investigate the inhibitory effects, a high‐content cell imaging system was employed to analyse HCC cells treated with HHT, YM155, Pano, IDA, and DNR. The results revealed a significant reduction in HCC cell viability, alongside a substantial increase in both early and late apoptotic indices. Specifically, IDA demonstrated the most profound effect on cell proliferation and early apoptosis, reducing cell counts by 4.18‐fold compared to the control and increasing the early apoptotic index by 14.40‐fold. HHT exerted the greatest impact on late apoptosis, which increased by 22.37‐fold relative to the control (Figure 1D,E). Following treatment with HHT, YM155, Pano, IDA, and DNR, both the size and number of HCC cell colonies were markedly diminished. Notably, DNR exhibited the most pronounced inhibitory effect on clonogenic survival, nearly abrogating visible colony formation and reducing colony numbers by 14.96‐fold compared to the control (Figure S1C,D). Together, classical drug screening identified clinical agents capable of effectively inhibiting the clonogenicity and proliferative potential of HCC cells in vitro.

### 3.2 Single‐Cell Screening Uncovered the Heterogeneous Transcriptional Response Characteristics

**FIGURE 2:**
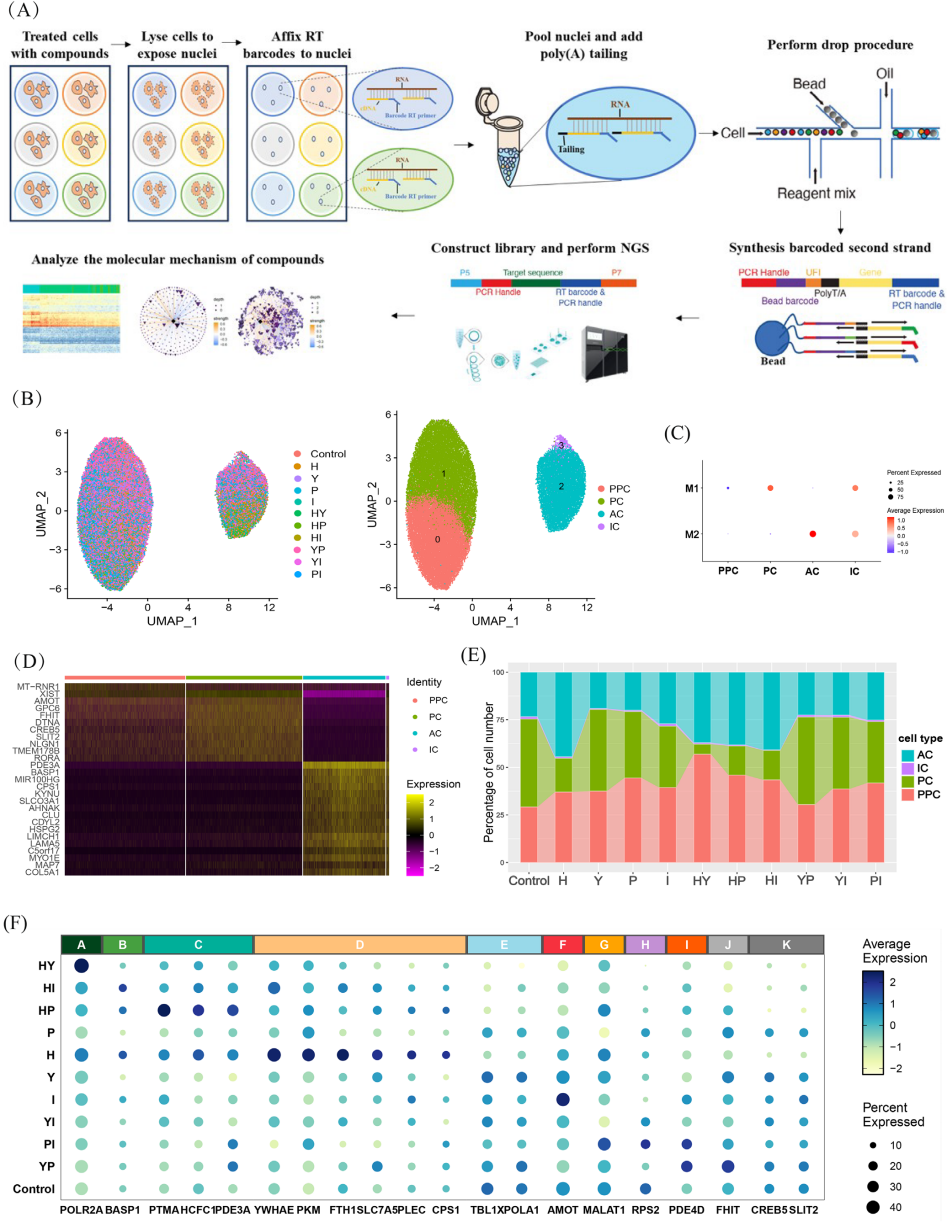
Single‐cell screening uncovered the heterogeneous transcriptional response characteristics. (A) Schematic of the basic workflow for snHH‐seq. (B) Uniform manifold approximation and projection (UMAP) embedding of the HCC cells analyzed in this study. Colour‐coded for different drug treatment (left) and cell type (right). (C) Dot plot showing representative module expression in each cluster of HCC cells. Average expression represents the average expression level of a specific gene within a defined cell population. Percent expressed refers to the proportion of cells within the given population that show detectable expression of the gene. (D) Heatmap showing representative gene expression in each cluster of HCC cells. (E) Bar chart showing the percentage of cell number in HCC cells with different drug treatment. (F) Dot plot showing representative gene expression in HCC cells with different drug treatment. Modules A‐K are defined based on the overall expression patterns of these 20 marker genes. Each module is composed of co‐expression patterns involving one or multiple genes. Average expression represents the average expression level of a specific gene within a defined cell population. Percent expressed refers to the proportion of cells within the given population that show detectable expression of the gene. H, HHT; Y, YM155; P, Pano; I, IDA; HY, HHT&YM155; HP, HHT&Pano; HI, HHT&IDA; YP, YM155&Pano; YI, YM155&IDA; PI, Pano&IDA; PPC, primary proliferative cells; PC, proliferation‐related cells; AC, apoptosis‐related cells; IC, intermediate cells.

Based on preliminary screening and functional experimental validation, HHT, YM155, Pano, IDA, and DNR were identified with significant inhibitory effects on the proliferation of HCC cells. Since both IDA and DNR are anthracycline drugs with similar mechanisms of action and metabolic pathways, and IDA exhibited slightly stronger inhibitory efficiency than DNR, only IDA was selected for subsequent drug combination screening. Utilizing the previously developed high‐throughput and high‐sensitive single‐cell sequencing platform snHH‐seq [27], a single experiment simultaneously analyzed the single‐cell transcriptomes of HCC cells treated with the following conditions: untreated control, HHT, YM155, Pano, IDA, HHT&YM155(HY), HHT&Pano(HP), HHT&IDA(HI), YM155&Pano(YP), YM155&IDA(YI), and Pano&IDA(PI) (Figure 2A). Following quality control and data preprocessing, a total of 46,901 cells were collected, with an average UMI count of 681.1 and an average gene count of 331.58 (Figure S2A,B, and Table S2). A key quality control measure was to set the mitochondrial proportion threshold of < 50% (Figure S2C). Recent evidence suggests that malignant tumor cells have elevated baseline mitochondrial gene expression, and high mitochondrial content often marks viable, functionally distinct subpopulations rather than low‐quality cells [32]. Consistent with this, our drug treatments on HCC cells induced cell death that was accompanied by a further increase in mitochondrial proportion.

Subsequently, unsupervised clustering analysis of these cells after dimensionality reduction identified four subpopulations (Figure 2B). Among them, cluster 1 (C1), defined as proliferation‐related cells (PC), was characterised by marker genes such as *GPC6*, *XIST*, *AMOT*, *SLIT2*, and *RPS2*. Cluster 2 (C2), defined as apoptosis‐related cells (AC), expressed genes such as *PDE3A*, *MIR100HG*, *FTH1*, and *SLC7A5*. Cluster 0 (C0) and cluster 3 (C3) lacked distinctive marker genes. Further hdWGCNA [30] co‐expression analysis of the single‐cell data revealed that the data could be divided into two modules: M1 and M2, corresponding to PC and AC, respectively. C0 did not belong to either module and was closer to C1, thus being defined as primary proliferative cells (PPC). C3, which exhibited low expression of both M1 and M2, was defined as intermediate cells (IC) (Figure 2C,D, and Figure S2D,E). Next, we analysed the changes in cell type proportions and cell cycle distribution across various treatment groups of HCC cells. Results revealed that drug treatments did not affect the cell types or cell cycle phases but significantly influenced the cell type proportions. Compared to the control and other treatment groups, the HY combination exhibited the most pronounced inhibitory effect. Specifically, the proportion of PC in HCC cells drastically decreased from 46.15% to 5.29%, while the proportion of AC increased from 23.42% to 37.04% (Figure 2E and Figure S2F).

Additionally, we conducted an analysis of transcriptional heterogeneity in HCC cells treated with individual drugs and drug combinations (Figure 2F). Results demonstrated that treatment with HHT and combination regimens HY, HI, and HP significantly suppressed the expression of *CREB5* and *SLIT2* in HCC cells (module K), aligning with existing studies identifying these genes as potential therapeutic targets in oncology [33, 34]. HHT specifically induced upregulation of genes including *YWHAE*, *PKM*, *FTH1*, *SLC7A5*, *PLEC*, and *CPS1* (module D), whereas such effects were not observed in the HY, HI, or HP combination. *AMOT* exhibits dual roles in tumorigenesis, either promoting or inhibiting tumour growth via modulation of signalling pathways such as Hippo and Wnt/β‐catenin [35]. Single‐cell data revealed that IDA markedly increased *AMOT* expression, whereas HY and HP combinations reduced its expression (module F), indicating that while these agents phenotypically suppress HCC growth, their underlying mechanisms exhibit distinct pathway divergences. Moreover, *POLR2A* is the largest subunit of RNA polymerase II, responsible for transcribing all protein‐coding genes. Dysfunction or dysregulated expression of *POLR2A* may disrupt transcriptional regulation in cancer cells. Liu et al. have found that in colorectal cancer, loss of *POLR2A* could lead to transcriptional dysregulation, thereby promoting tumour progression [36]. In our study, HY treatment was observed to upregulate *POLR2A* expression (module A), though the mechanisms require further exploration. In summary, single‐cell sequencing technology identified the transcriptional heterogeneity of HCC cells in response to drug treatments and screened out the drug combination HY with the most potent inhibitory efficiency.

### 3.3 Functional Experiments Validated the Inhibitory Efficiency of Drug Combination

**FIGURE 3:**
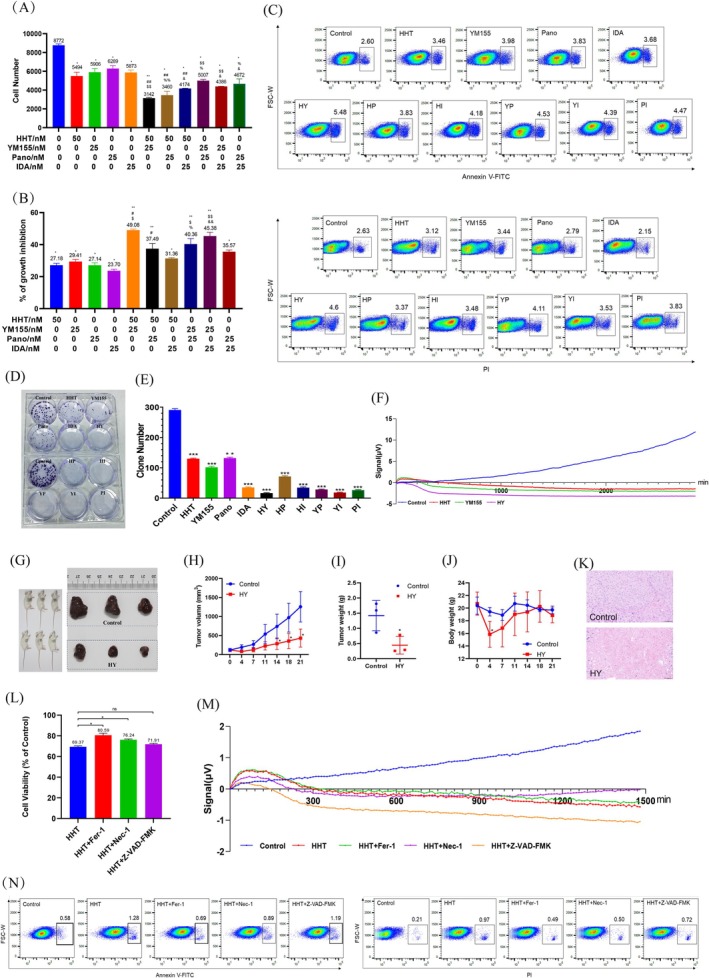
Functional experiments validated the inhibitory efficiency of drug combination. (A) Cell number of different combinations of drugs against HepG2 cell line. Cells were seeded in a 96‐well plate for 48 h, and the cell number was detected by an Operetta CLS HCS reader. (B) Cell growth inhibition percentage (%) of different combinations of drugs against HepG2 cell line. Cells were seeded in a 96‐well plate for 48 h, and the cell growth inhibition percentage (%) was detected with CCK‐8 kit. (C) Early apoptosis percentage (top) and late apoptosis percentage (bottom) of different combinations of drugs against HepG2 cell line. Cells were seeded with different drug combinations in a 6‐well plate for 48 h, and the Annexin V‐FITC and PI expression was detected by LSR Fortessa flow cytometry. (D) Colony formation assay was conducted to investigate tumour growth after treatment with different drug combinations for 14 days. The colonies were visualised with the images. (E) The corresponding histogram showed the colony numbers. (F) Real‐time signal analysis of cell proliferation after treatment with HHT, YM155, and HHT&YM155. (G) Images of tumours from control and HY‐treated mice groups. (H) Statistical analysis of tumour volumes of mice in the control and HY treatment groups along with time. (I) Statistical analysis of tumour weight of mice in the control and HY treatment groups. (J) Statistical analysis of body weight of mice in the control and HY treatment groups along with time. (K) Representative images of the HE staining in tumour sections, scale bar: 50 μM. (L) Cell viability percentage (%) of HHT, HHT&Fer‐1, HHT&Nec‐1, and HHT&Z‐VAD‐FMK against HepG2 cell line. Cells were seeded in a 96‐well plate for 48 h, and the cell viability percentage was detected with CCK‐8 kit. (M) Real‐time signal analysis of cell proliferation after treatment with HHT, HHT&Fer‐1, HHT&Nec‐1, and HHT&Z‐VAD‐FMK. (N) Early apoptosis percentage (left) and late apoptosis percentage (right) of HHT, HHT&Fer‐1, HHT&Nec‐1, and HHT&Z‐VAD‐FMK against HepG2 cell line. Cells were seeded with different drug combinations in a 6‐well plate for 48 h, and the Annexin V‐FITC and PI expression was detected by LSR Fortessa flow cytometry. H, HHT; Y, YM155; P, Pano; I, IDA; HY, HHT&YM155; HP, HHT&Pano; HI, HHT&IDA; YP, YM155&Pano; YI, YM155&IDA; PI, Pano&IDA. Data were presented as mean ± SD (*n* = 3) and comparisons were performed with unpaired two‐tailed Student's t‐test. *p < 0.05, **p < 0.01, ***p < 0.001 versus negative control group; ^#^ *p* < 0.05, ^##^ *p* < 0.01 versus HHT alone group; ^$^ *p* < 0.05, ^$$^ *p* < 0.01 versus YM155 alone group; ^%^ *p* < 0.05, ^%%^ *p* < 0.01 versus Pano alone group; ^&^ *p* < 0.05, ^&&^ *p* < 0.01 versus IDA alone group, ns, no significance.

To validate the drug combinations' inhibitory efficiency identified through single‐cell sequencing, we further conducted functional experiments. High‐content statistical analysis and cell viability assays confirmed that the pairwise combinations of HHT, YM155, Pano, and IDA exhibited significantly greater efficacy than individual drugs (Figure 3A,B). Notably, the combination of HY exhibited the best performance, with a growth inhibition rate 1.8 times higher than HHT alone and 1.7 times higher than YM155 alone, indicating a strong synergistic inhibitory effect (Figure 3B). Flow cytometry analysis also confirmed that the drug combinations were significantly more effective than individual drugs. In early and late apoptosis, the HY showed the strongest inhibitory effects. Specifically, in early apoptosis, the apoptotic ratio of the HY was 1.58 times higher than HHT and 1.38 times higher than YM155. In late apoptosis, the proportions were 3.12% for HHT, 3.44% for YM155, and 4.6% for the HY, demonstrating significant synergistic inhibition (Figure 3C). In the colony formation assay, the HY remained the most effective, reducing colony formation by 7.88 times compared to HHT and by 6.15 times compared to YM155 (Figure 3D,E). These in vitro experimental results demonstrate that the drug combinations exhibit significantly greater cytotoxicity and anti‐proliferative activity against HCC cells compared to individual drugs. Notably, the HY performed optimally. This finding was corroborated by real‐time cell analysis (RTCA) impedance‐based assays, where the proliferation signals were most markedly reduced in the HY combination compared to HHT or YM155 alone (Figure 3F).

To further investigate the anti‐tumour efficacy of the HY, we conducted in vivo xenograft studies. Results showed a significant reduction in tumour burden, including tumour volume and tumour weight, following treatment with the HY compound combination. A statistically significant difference in tumour volume was observed after 14 days, while no significant changes were noted in body weight among the mice (Figure 3G–J). Haematoxylin and eosin staining revealed well‐preserved nuclear structure, deep staining, and uniform cell distribution with rare necrotic areas in the control group. In contrast, the HY‐treated HCC cells exhibited enlarged and deformed nuclei, indistinct cellular boundaries, and an expanded necrotic region within the tumour tissue (Figure 3K). Consistent with the in vitro findings, the in vivo studies further demonstrated that the HY drug combination effectively inhibited HCC tumour growth.

In our single‐cell sequencing analysis of gene expression in response to drug treatments, we found that the effects of compounds in HCC changed the expression level of Ferritin Heavy Chain 1 (*FTH1*), suggesting the action of the drug may be related to ferroptosis (Figure 2F). To validate this, we co‐treated HCC cultures with HHT or YM155 and inhibitors targeting distinct cell death pathways: Fer‐1 (ferroptosis inhibitor), Nec‐1 (necroptosis inhibitor), and Z‐VAD‐FMK (apoptosis inhibitor). The results of cell viability assays showed that treatment with Fer‐1 (100 nM) significantly rescued HCC cells from death induced by HHT (16.18%) or YM155 (6.19%) (Figure 3L and Figure S3A). Similarly, real‐time cell analysis (RTCA) impedance‐based proliferation assays demonstrated that proliferation rates were significantly restored in Fer‐1 co‐treated groups (Figure 3M and Figure S3B). Interestingly, in HHT or YM155‐treated HCC cells, Fer‐1 had the greatest inhibitory effect on early and late apoptosis, whereas Z‐VAD‐FMK had the least inhibitory effect (Figure 3N and Figure S3C). Collectively, these results suggest that HHT and YM155 may trigger ancillary apoptotic signalling; their primary anti‐proliferative effect in HCC is mediated through ferroptosis, as evidenced by the selective rescue with Fer‐1. This underscores ferroptosis induction as a key mechanism of action for these compounds.

### 3.4 Molecular Mechanisms Elucidation at the Single‐Cell Level

**FIGURE 4:**
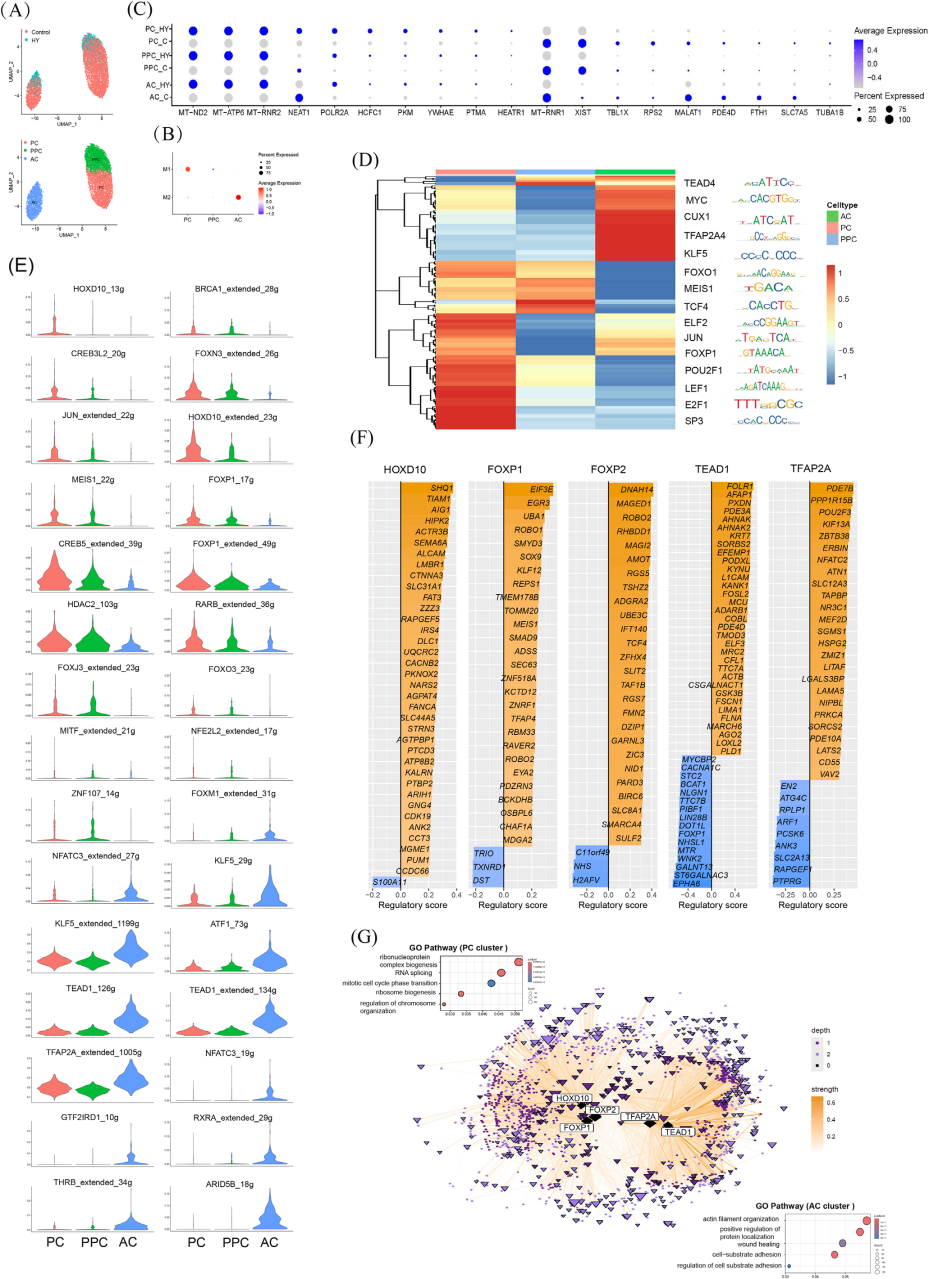
Molecular mechanisms elucidation at the single‐cell level. (A) UMAP embedding of the HCC cells analysed in this study. Colour‐coded for control and HY treatment (top) and cell type (bottom). (B) Dot plot showing representative module expression in each cluster of HCC cells with HY treatment. Average expression represents the average expression level of a specific gene within a defined cell population. Percent expressed refers to the proportion of cells within the given population that show detectable expression of the gene. (C) Dot plot showing representative gene expression in each cell type with HY treatment. Average expression represents the average expression level of a specific gene within a defined cell population. Percent expressed refers to the proportion of cells within the given population that show detectable expression of the gene. (D) Heatmap showing the activity distribution of each regulon in HCC cells with HY treatment. (E) Violin plot showing representative regulon in each cluster of HCC cells with HY treatment. (F) Bar plot showing the top predicted target genes for each TF, split by whether the target gene was positively (right) or negatively (left) correlated with the TF based on gene expression. The regulatory importance score from XGBoost is plotted on the x‐axis, and target genes are ranked by their importance scores. (G) The TF network showing regulatory links originating from our TF of interest in (F). The nodes represent TFs and genes, and the edges represent inferred regulatory relationships. The selected TF is shown as a diamond, other TFs are shown as triangles, and genes are shown as circles. The size of each node corresponds to the outdegree in the network. The colour of the edges represents the strength of the TF‐gene interaction based on the Pearson correlation of gene expression. The colour of each node represents the number of links to the selected TFs. GO enrichment analysis of differentially expressed genes in PC (left) and AC (right) subpopulation. HY, HHT&YM155; PPC, primary proliferative cells; PC, proliferation‐related cells; AC, apoptosis‐related cells.

Through comprehensive in vitro and in vivo functional validation, we confirmed that the HY drug combination effectively inhibits the proliferation of HCC cells. To elucidate its mechanisms basis at single‐cell resolution, we performed UMAP clustering and co‐expression analysis on single‐cell data from control and HY‐treated samples, revealing three transcriptionally distinct subclusters: PC, AC, and PPC. These subclusters aligned with two co‐expression modules: M1 (enriched in PC), and M2 (enriched in AC), while PPC lacked module association (Figure 4A,B and Figure S4C). Additionally, differential gene expression analysis across subclusters demonstrated that HY treatment universally upregulated apoptosis‐related genes (e.g., *MT‐ND2*, *MT‐ATP6*) and downregulated proliferation‐related genes (e.g., *XIST*, *RPS2*). Interestingly, HY induced opposing expression trends in mitochondrial ribosomal genes: *MT‐RNR1* (a core component of the mitochondrial small subunit) was suppressed, while *MT‐RNR2* (a structural component of the large subunit) was elevated, and the specific mechanism requires further exploration. HY treatment also selectively inhibited the expression of lncRNA *NEAT1* in the AC and PPC populations, but not in the PC population. This is consistent with existing research that *NEAT1* regulates miR‐362‐3p and MIOX in the ferroptosis signalling pathway in liver tumours [37] (Figure 4C), further supporting ferroptosis as a key HY‐driven mechanism. Moreover, by characterising signature genes associated with ferroptosis‐related lipid metabolism pathways and iron homeostasis, we found that HY treatment of HCC cells downregulates the expression of genes such as glutathione peroxidase 4 (*GPX4*) [38], dihydroorotate dehydrogenase (*DHODH*) [39], and transferrin receptor (*TFRC*) [40], while upregulating ACSL4, a critical inducer of ferroptosis [41] (Figure S4A). This dysregulation exhibited distinct, cell‐type‐specific patterns (Figure S4B). The suppression of *GPX4* was a consistent response observed across all three cell types (AC, PPC, PC). *TFRC* was most significantly downregulated in AC, while *SNX5*, *USP7*, and *SLC3A2* showed greater downregulation in PC. *ACSL4* upregulation was specific to PPC and PC. Although HY treatment modulates the expression of key ferroptosis‐related genes in a cell‐type‐specific manner, the overall pathway enrichment shift for the core ferroptosis pathway was subtle. Together, these single‐cell insights reveal that HY coordinately triggers apoptosis, inhibits proliferation, and dysregulates mitochondrial and ferroptosis‐associated networks, with subpopulation‐specific effects shaping its therapeutic efficacy.

To delineate the transcriptional mechanism underlying HY‐treated effects, we constructed a gene regulatory network (GRN) from single‐cell data of HY‐treated HCC cells using SCENIC [29]. The transcription factor (TF) regulon activity heatmap displayed the activity distribution and clustering patterns of each regulon (Figure 4D, Figure S4D and Table S3). Different TF regulons showed distinct regulatory patterns: HOXD10 exclusively regulated the PC cluster; BRCA1, CREB3L2, JUN, and MEIS1 co‐regulated both PC and PPC clusters; CREB5, FOXP1, HDAC2, and RARB primarily governed PC/PPC clusters with minimal regulation of the AC cluster; KLF5, ATF1, TEAD1, and TFAP2A mainly targeted the AC cluster while retaining influence on PC/PPC clusters; whereas NFATC3, GTF2IRD1, RXRA, THRB, and ARID5B specifically regulated the AC cluster (Figure 4E and Figure S4E). Within this network, we observed key ferroptosis‐related TFs with cell‐type‐specific activity. These included TFAP2A [42], and KLF5 [43] in AC, TCF4 [44] and ATF4 [45] in PPC, E2F1 [46] in PC, as well as BRCA1 [47] in both PPC and PC. Notably, BRCA1 and E2F1 are known to play a context‐dependent dual role in ferroptosis regulation. GO analysis of their target genes (Table S3) revealed a strong association with cell cycle processes: 28 targets of BRCA1 were enriched in the “regulation of G2/M transition of mitotic cell cycle” (GO:0010389), while 986 targets of E2F1 were enriched in “mitotic cell cycle” (GO:0000278). Given that cell cycle arrest has a potent suppressive effect on ferroptosis [48], HY‐induced cell cycle progression may counteract ferroptosis defense mechanisms by modulating the activity of these dual‐role TFs in PC and PPC. That is HY may activate a compensatory, pro‐ferroptotic program in PC and PPC by modulating dual‐role TFs linked to cell cycle progression.

Further functional annotation of regulon targets highlighted bifurcated effects: HOXD10, FOXP1, and FOXP2 regulated multiple tumour proliferation‐related genes (e.g., *SOX9*, *MEIS1*, *AMOT*), while TEAD1 and TFAP2A controlled apoptosis‐associated genes (e.g., *PDE3A*, *KYNU*, *POU2F3*) (Figure 4F). Network topology analysis further exhibited dynamic crosstalk and differential regulatory intensities between regulons, reflecting the complexity of HY‐induced GRN in HCC cells (Figure 4G). The regulatory network is divided into two main components. On the left, the focus is on the PC cluster, where GO enrichment analysis reveals associations with pathways such as ribonucleoprotein complex biogenesis and ribosome biogenesis. On the right, the analysis centres on the AC subpopulation, with GO enrichment highlighting pathways including positive regulation of protein localization and wound healing.

Notably, differential regulon analysis identified JUN as the predominant regulator mediating HY‐induced proliferation inhibition, predominantly active in the AC cluster, consistent with differential gene expression patterns (Figure S4F). JUN formed intricate networks with MYC, POU2F1, RORA, and other regulators to amplify HCC cell proliferation arrest (Figure S4G). To validate this, we administered the JUN‐specific inhibitor T‐5224 (10 μM) in HCC cells following HY‐mediated suppression of their in vitro proliferation. Cell viability assays demonstrated that T‐5224 effectively rescued cell death induced by HY, achieving a rescue rate of 31.99% (Figure S4H). Similarly, flow cytometric analysis revealed that T‐5224 treatment most effectively counteracted HY‐induced early and late apoptosis in HCC cells (Figure S4I). These experimental findings corroborate the single‐cell RNA sequencing data, collectively indicating that JUN mediates the inhibitory effect of HY on the proliferation of HCC cells. Collectively, our GRN analysis delineated key cluster‐specific regulons underlying HY's effects on HCC cells, providing a foundation for precision diagnosis and treatment in clinical hepatology.

## 4 Discussion

In this study, we employed high‐throughput single‐cell screening and functional validation assays to demonstrate that the novel combination therapy HY (HHT&YM155) effectively inhibits HCC cell proliferation, with mechanistic insights elucidated at the single‐cell level. While HHT, a ribosome inhibitor, is known to induce the rapid turnover of several key oncoproteins (e.g., c‐MYC, MCL‐1) and potently triggers apoptosis in leukaemia cells, it exhibits limited efficacy against solid tumour cells [49, 50]. Qin et al. [51] demonstrated that activation of the JNK‐USP36‐Snail1 axis drives HHT resistance in solid tumours and that combinatorial inhibition of this axis synergizes with HHT to inhibit solid tumour proliferation and migration. In our data, single‐cell transcriptomic analyses and functional experiments consistently confirm that the drug combinations achieve significantly greater therapeutic efficacy than either drug alone. Notably, our findings reveal that conventional haematological malignancies drugs (e.g., AZA, AraC, VEN, Rux) exhibit markedly reduced efficacy in HCC cells, which may be due to substantial intrinsic molecular distinctions between haematopoietic tumours and solid tumours [52].

The snHH‐seq platform offers distinct advantages in drug screening by: (1) transcending conventional single‐phenotype assessments through leveraging single‐cell resolution to uncover molecular mechanisms of drug action; (2) enabling parallel analysis across multiple time points and dosage conditions via pre‐index technology; and (3) establishing correlations between short‐term transcriptional responses and long‐term cellular viability. Additionally, this platform employs a random primer strategy during reverse transcription to capture total transcriptomes at single‐cell resolution. This allows for comprehensive analysis of transcriptional changes in response to drug treatment at a single‐cell level. Our snHH‐seq analysis identified multiple lncRNAs functionally associated with HY treatment response in HCC. For instance, *NEAT1*, a well‐characterised oncogenic lncRNA [53], exhibited cluster‐specific regulation post‐HY treatment, with marked upregulation in the AC cluster but downregulation in the PC cluster, suggesting its potential involvement in cell death. This aligns with recent findings demonstrating that *NEAT1's* tumour‐suppressive function in acute myeloid leukaemia (AML) [54]. *MALAT1*, an abundant, evolutionarily conserved ~7 to 8‐kb lncRNA localised to nuclear speckles and overexpressed in cancers, has long been associated with poor prognosis and metastasis across malignancies [55]. However, its oncogenic versus tumour‐suppressive roles remain contentious. Our single‐cell data revealed that HY significantly suppresses *MALAT1* expression, particularly in the AC cluster, supporting its potential oncogenic role in HCC progression. Furthermore, additional lncRNA candidates including *YWHAE*, *XIST*, and *LIPE‐AS1* emerged as promising precision targets for HCC therapy. These findings, coupled with the emerging understanding of the complex roles of lncRNAs in cancer biology, highlight the importance of studying lncRNA regulation in drug response for developing next‐generation anticancer therapies.

The vast amount of data generated by high‐throughput single‐cell sequencing demands the power of deep learning to unlock its full potential. In future studies, we might leverage artificial intelligence to further delve into the data presented in this study, identifying more precise commonalities in drug mechanisms and cell‐type‐specific response patterns, thereby providing robust data support for mechanistic elucidation and predictive modeling of therapeutic efficacy.

## Author Contributions

Mengmeng Jiang, Haide Chen, Wenzhao Zhou, Bin Xu, Tingyue Zhang, Guangyan Li, and Junqing Wu were responsible for the development of methodology, analysis, and experiments. Mengmeng Jiang, Guoxia Wen, and Yuqing Mei performed the bioinformatics analysis. Jingjing Wang, Xiaoping Han, Xudong Fu, and Guoji Guo conceptualised and oversaw all phases of the study, including experimental design, execution, and analytical workflows. All authors read and approved the final manuscript.

## Funding

This work was supported by Zhejiang Provincial Natural Science Foundation of China, LTGC23C070002, LTGC24B050001, LQ24H080006; the National Natural Science Foundation of China, 82090012, 82400178, 32570784.

## Ethics Statement

All animal care and animal experiments in this study were performed in accordance with China's National Legislation and the institutional ethical guidelines. Female NCG mice were purchased from GemPharmatech Co. Ltd. (Jiangsu, China). Mice were maintained in individual cages at a room temperature of 22 ± 2°C and humidity of 50%–60%, on a 12:12 light–dark cycle. 5 × 10^5^ cells were subcutaneously inoculated into the right scruff of each mouse (*n* = 3/group). On day 3 after inoculation, mice were intraperitoneally (i.p.) injected with DMSO (5% v/v) or with HHT (2 mg/kg) and YM155 (2 mg/kg) daily. Mice were monitored for tumour size daily and sacrificed on the indicated day after i.p. Tumour weight, volume, and photos were taken. The xenograft tumour samples were subjected to HE straining analyses. Tumour size was measured with a calliper and tumour volume was calculated by width^2^ × length × 1/2.

## Conflicts of Interest

The authors declare no conflicts of interest.

## Supporting information


**Figure S1:** Drug screening identified compounds that effectively inhibit HCC cell proliferation. (A) Images of HepG2 cell morphology. (B) Cell viability percentage (%) of AZA, AraC, VEN, and Rux against HepG2 cell line. Cells were seeded in a 96‐well plate and treated with 1.25 μM, 2.5 μM, 5 μM, 10 μM drug for 48 h, and the cell viability percentage was detected with CCK‐8 kit. (C) Colony formation assay was conducted to investigate tumour growth after treatment with HHT, YM155, Pano, IDA, and DNR for 14 days. The colonies were visualised with the images. (D) The corresponding histogram showed the colony numbers. Data were presented as mean ± SD (*n* = 3) and comparisons were performed with unpaired two‐tailed Student's t test. *p < 0.05. The absence of a * mark indicates no statistical significance.
**Figure S2:** Single‐cell screening uncovered the heterogeneous transcriptional response characteristics. (A) UMAP embedding of the HCC cells analysed in this study. Colour‐coded for specific drug treatment (left) and cell type (right). (B) UMAP embedding of the HCC cells analysed in this study. Colour‐coded for RT barcode. (C) The violin plot of chrMT% distribution. (D) HdWGCNA analysis of HCC cells with different drug treatment identified two modules. (E) UMAP embedding of the HCC cells analysed in this study. Colour‐coded for module 1 (left) and module 2 (right). (F) Bar chart showing the percentage of cell cycle in HCC cells with different drug treatment. H, HHT; Y, YM155; P, Pano; I, IDA; HY, HHT&YM155; HP, HHT&Pano; HI, HHT&IDA; YP, YM155&Pano; YI, YM155&IDA; PI, Pano&IDA; PPC, primary proliferative cells; PC, proliferation‐related cells; AC, apoptosis‐related cells; IC, intermediate cells.
**Figure S3:** Functional experiments validated the inhibitory efficiency of drug combination. (A) Cell viability percentage (%) of YM155, YM155&Fer‐1, YM155&Nec‐1, and YM155&Z‐VAD‐FMK against HepG2 cell line. Cells were seeded in 96‐well plate for 48 h, and the cell viability percentage was detected with CCK‐8 kit. (B) Real‐time signal analysis of cell proliferation after treatment with YM155, YM155&Fer‐1, YM155&Nec‐1, and YM155&Z‐VAD‐FMK. (C) Early apoptosis percentage (top) and late apoptosis percentage (bottom) of YM155, YM155&Fer‐1, YM155&Nec‐1, and YM155&Z‐VAD‐FMK against HepG2 cell line. Cells were seeded with different drug combination in 6‐well plate for 48 h, and the Annexin V‐FITC and PI expression was detected by LSR Fortessa flow cytometry. Data were presented as mean ± SD (*n* = 3) and comparisons were performed with unpaired two‐tailed Student's t test. *p < 0.05, ns, no significance.
**Figure S4:** Molecular mechanisms elucidation at the single‐cell level. (A) Dot plot showing the expression of signature genes associated with ferroptosis in HCC with (HY) or without (Control) HY treatment. Average expression represents the average expression level of a specific gene within a defined cell population. Percent expressed refers to the proportion of cells within the given population that show detectable expression of the gene. (B) Dot plot showing the expression of signature genes associated with ferroptosis across different cell types with (HY) or without (C) HY treatment. Average expression represents the average expression level of a specific gene within a defined cell population. Percent expressed refers to the proportion of cells within the given population that show detectable expression of the gene. (C)UMAP embedding of the HCC cells with HY treatment. Colour‐coded for module 1 (left) and module 2 (right). (D) Bar chart showing the number of cells per regulon (left) and the number of regulons per cell (right). (E) Dot plot showing the representative regulon in each cluster of HCC cells with HHT treatment. Regulon specificity score (RSS) measures the specificity score of a regulon across different cell types. Z score assesses the expression level of an individual gene relative to its background distribution, measured in standard deviations. (F) Scatter plot showing the effect sizes from the differential regulon test for the positive (x‐axis) and negative (y‐axis) regulons. For the TFs in the top left corner, the negatively‐correlated target genes are up‐regulated of AC cluster in HY treatment relative to control (left), and the negatively‐correlated target genes are up‐regulated of in HY treatment relative to control (right). Each point represents a TF, coloured by the module assignment in (C). Diamonds represent TFs that are also significantly differentially expressed, while circles are not differentially expressed. TFs that did not reach significance are opaque while the significant TFs have a black outline. The number of significantly differentially expressed regulons in each quadrant of the plot are labelled in the corners. (G) The TF network showing regulatory links originating from JUN. The nodes represent TFs and genes, and the edges represent inferred regulatory relationships. The selected TF is shown as a diamond, other TFs are shown as triangles, and genes are shown as circles. The size of each node corresponds to the outdegree in the network. The colour of the edges represents the strength of the TF‐gene interaction based on the pearson correlation of gene expression. The colour of each node represents the number of links to the selected TFs. (H) Cell viability percentage (%) of HY and HY + T5224 against HepG2 cell line. Cells were seeded in a 96‐well plate for 48 h, and the cell viability percentage was detected with CCK‐8 kit. (I) Early apoptosis percentage (upper) and late apoptosis percentage (below) of HY and HY + T5224 against HepG2 cell line. Cells were seeded with different drug combination in 6‐well plate for 48 h, and the Annexin V‐FITC and PI expression was detected by LSR Fortessa flow cytometry. HY, HHT&YM155; PPC, primary proliferative cells; PC, proliferation‐related cells; AC, apoptosis‐related cells. Data were presented as mean ± SD (*n* = 3) and comparisons were performed with unpaired two‐tailed Student's t‐test. *p < 0.05, **p < 0.01, ***p < 0.001.

**Table S1:** Drug screening identified compounds that effectively inhibit HCC cell proliferation. The information of drugs used in this study, including full name, abbreviation, specification, solvent and aliquot concentration.

**Table S2:** Single‐cell screening uncovered the heterogeneous transcriptional response characteristics. (Sheet 1) The RT barcode information corresponding to each drug treatment group. (Sheet 2) The cell number of each cell type information corresponding to each drug treatment group. (Sheet 3) The cell number of each cell cycle information corresponding to each drug treatment group. (Sheet 4–14) Differentially expressed genes detected in each cell type for HCC cells with different drug treatment. Yellow labels indicate specific marker genes of cell clusters. Genes are selected by log foldchange > 0.25, Bonferroni‐adjusted *p*‐value < 0.1, expressed in at least 15% of cells in either population (Seurat FindAllMarkers). Log fold change is calculated as arithmetic mean of log10 cpm values of one population minus the arithmetic mean of log10 cpm values of the second, and fold change is 10log_foldchange. *P*‐values were calculated by the Wilcoxon rank sum test.

**Table S3:** Molecular mechanisms elucidation at the single‐cell level. (Sheet 1–3) Differentially expressed genes detected in each cell cluster for HCC cells with HY treatment. Genes are selected by log foldchange > 0.25, Bonferroni‐adjusted *p*‐value < 0.1, expressed in at least 15% of cells in either population (Seurat FindAllMarkers). Log fold change is calculated as arithmetic mean of log10 cpm values of one population minus the arithmetic mean of log10 cpm values of the second, and fold change is 10log_foldchange. *p*‐Values were calculated by the Wilcoxon rank sum test. (Sheet 4) The information of hub genes in HCC cells with HY treatment. (Sheet 5) The information of enriched motif in HCC cells with HY treatment. (Sheet 6) The information of regulon and target genes in HCC cells with HY treatment. (Sheet 7) The information of AUC cell thresholds in HCC cells with HY treatment.

**Data S1:** Supporting Information.

## Data Availability

The data that support the findings of this study are openly available in GSE291757 at https://www.ncbi.nlm.nih.gov/geo/.

## References

[1] B. Van de Sande , J. S. Lee , E. Mutasa‐Gottgens , et al., “Applications of Single‐Cell RNA Sequencing in Drug Discovery and Development,” Nature Reviews. Drug Discovery 22 (2023): 496–520. 37117846 10.1038/s41573-023-00688-4 PMC10141847

[2] J. Wang , F. Ye , H. Chai , et al., “Advances and Applications in Single‐Cell and Spatial Genomics,” Science China. Life Sciences 68 (2024): 1226–1282. 39792333 10.1007/s11427-024-2770-x

[3] A. Baysoy , Z. Bai , R. Satija , and R. Fan , “The Technological Landscape and Applications of Single‐Cell Multi‐Omics,” Nature Reviews. Molecular Cell Biology 24 (2023): 695–713. 37280296 10.1038/s41580-023-00615-w PMC10242609

[4] M. T. Chang , F. Shanahan , T. T. T. Nguyen , et al., “Identifying Transcriptional Programs Underlying Cancer Drug Response With TraCe‐Seq,” Nature Biotechnology 40 (2022): 86–93. 10.1038/s41587-021-01005-3 34531539

[5] X. Han , R. Wang , Y. Zhou , et al., “Mapping the Mouse Cell Atlas by Microwell‐Seq,” Cell 172 (2018): 1091–1107. 29474909 10.1016/j.cell.2018.02.001

[6] X. Han , Z. Zhou , L. Fei , et al., “Construction of a Human Cell Landscape at Single‐Cell Level,” Nature 581 (2020): 303–309. 32214235 10.1038/s41586-020-2157-4

[7] J. Hao , A. Ma , L. Wang , et al., “General Requirements for Stem Cells,” Cell Proliferation 53 (2020): e12926. 33146418 10.1111/cpr.12926 PMC7705904

[8] M. Jiang , Y. Xiao , E. Weigao , et al., “Characterization of the Zebrafish Cell Landscape at Single‐Cell Resolution,” Frontiers in Cell and Developmental Biology 9 (2021): 743421. 34660600 10.3389/fcell.2021.743421 PMC8517238

[9] C. Meiller , F. Montagne , T. Z. Hirsch , et al., “Multi‐Site Tumor Sampling Highlights Molecular Intra‐Tumor Heterogeneity in Malignant Pleural Mesothelioma,” Genome Medicine 13 (2021): 113. 34261524 10.1186/s13073-021-00931-w PMC8281651

[10] F. Ye , J. Wang , J. Li , Y. Mei , and G. Guo , “Mapping Cell Atlases at the Single‐Cell Level,” Advanced Science (Weinheim, Germany) 11 (2024): e2305449. 10.1002/advs.202305449 PMC10885669 38145338

[11] J. M. McFarland , B. R. Paolella , A. Warren , et al., “Multiplexed Single‐Cell Transcriptional Response Profiling to Define Cancer Vulnerabilities and Therapeutic Mechanism of Action,” Nature Communications 11 (2020): 4296. 10.1038/s41467-020-17440-w PMC7453022 32855387

[12] S. R. Srivatsan , J. L. McFaline‐Figueroa , V. Ramani , et al., “Massively Multiplex Chemical Transcriptomics at Single‐Cell Resolution,” Science 367 (2020): 45–51. 31806696 10.1126/science.aax6234 PMC7289078

[13] Z. Lin , F. Wang , R. Yin , et al., “Single‐Cell RNA Sequencing and Immune Microenvironment Analysis Reveal PLOD2‐Driven Malignant Transformation in Cervical Cancer,” Frontiers in Immunology 15 (2024): 1522655. 39840054 10.3389/fimmu.2024.1522655 PMC11747275

[14] X. Feng , Z. Luo , W. Zhang , et al., “Zn‐DHM Nanozymes Enhance Muscle Regeneration Through ROS Scavenging and Macrophage Polarization in Volumetric Muscle Loss Revealed by Single‐Cell Profiling,” Advanced Functional Materials 35 (2025): 2506476.

[15] S. Gallage , M. Garcia‐Beccaria , M. Szydlowska , et al., “The Therapeutic Landscape of Hepatocellular Carcinoma,” Medicus 2 (2021): 505–552. 10.1016/j.medj.2021.03.002 35590232

[16] Z. Peng , J. Wu , S. Hu , et al., “Requirments for Primary Human Hepatocyte,” Cell Proliferation 55 (2022): e13147. 34936148 10.1111/cpr.13147 PMC9055892

[17] R. L. Siegel , K. D. Miller , N. S. Wagle , and A. Jemal , “Cancer Statistics, 2023,” CA: A Cancer Journal for Clinicians 73 (2023): 17–48. 36633525 10.3322/caac.21763

[18] H. Jin , L. Wang , and R. Bernards , “Rational Combinations of Targeted Cancer Therapies: Background, Advances and Challenges,” Nature Reviews. Drug Discovery 22 (2023): 213–234. 36509911 10.1038/s41573-022-00615-z

[19] Q. Wu , W. Qian , X. Sun , and S. Jiang , “Small‐Molecule Inhibitors, Immune Checkpoint Inhibitors, and More: FDA‐Approved Novel Therapeutic Drugs for Solid Tumors From 1991 to 2021,” Journal of Hematology & Oncology 15 (2022): 143. 36209184 10.1186/s13045-022-01362-9 PMC9548212

[20] D. Li , L. Xu , J. Ji , et al., “Sintilimab Combined With Apatinib Plus Capecitabine in the Treatment of Unresectable Hepatocellular Carcinoma: A Prospective, Open‐Label, Single‐Arm, Phase II Clinical Study,” Frontiers in Immunology 13 (2022): 944062. 36091003 10.3389/fimmu.2022.944062 PMC9459134

[21] S. Qin , S. L. Chan , S. Gu , et al., “Camrelizumab Plus Rivoceranib Versus Sorafenib as First‐Line Therapy for Unresectable Hepatocellular Carcinoma (CARES‐310): A Randomised, Open‐Label, International Phase 3 Study,” Lancet 402 (2023): 1133–1146. 37499670 10.1016/S0140-6736(23)00961-3

[22] X. D. Zhu , C. Huang , Y. H. Shen , et al., “Downstaging and Resection of Initially Unresectable Hepatocellular Carcinoma With Tyrosine Kinase Inhibitor and Anti‐PD‐1 Antibody Combinations,” Liver Cancer 10 (2021): 320–329. 34414120 10.1159/000514313 PMC8339461

[23] X. Nan , B. Zhang , J. Hao , et al., “Requirements for Human Haematopoietic Stem/Progenitor Cells,” Cell Proliferation 55 (2022): e13152. 34936155 10.1111/cpr.13152 PMC9055899

[24] F. Raza , M. Zheng , H. Zhong , et al., “Engineered Tumor Microvesicles Modified by SP94 Peptide for Arsenic Trioxide Targeting Drug Delivery in Liver Cancer Therapy,” Biomaterials Advances 155 (2023): 213683. 37925825 10.1016/j.bioadv.2023.213683

[25] L. Wang , S. Bi , Z. Li , et al., “Napabucasin Deactivates STAT3 and Promotes Mitoxantrone‐Mediated cGAS‐STING Activation for Hepatocellular Carcinoma Chemo‐Immunotherapy,” Biomaterials 313 (2025): 122766. 39180916 10.1016/j.biomaterials.2024.122766

[26] Y. Sun , L. Wu , Y. Zhong , et al., “Single‐Cell Landscape of the Ecosystem in Early‐Relapse Hepatocellular Carcinoma,” Cell 184 (2021): 404–421. 33357445 10.1016/j.cell.2020.11.041

[27] H. Chen , X. Fang , J. Shao , et al., “Pan‐Cancer Single‐Nucleus Total RNA Sequencing Using snHH‐Seq,” Advanced Science (Weinheim, Germany) 11 (2024): e2304755. 10.1002/advs.202304755 PMC10837386 38010945

[28] R. Satija , J. A. Farrell , D. Gennert , A. F. Schier , and A. Regev , “Spatial Reconstruction of Single‐Cell Gene Expression Data,” Nature Biotechnology 33 (2015): 495–502. 10.1038/nbt.3192 PMC4430369 25867923

[29] B. Van de Sande , C. Flerin , K. Davie , et al., “A Scalable SCENIC Workflow for Single‐Cell Gene Regulatory Network Analysis,” Nature Protocols 15 (2020): 2247–2276. 32561888 10.1038/s41596-020-0336-2

[30] J. E. Childs , S. Morabito , S. Das , et al., “Relapse to Cocaine Seeking Is Regulated by Medial Habenula NR4A2/NURR1 in Mice,” Cell Reports 43 (2024): 113956. 38489267 10.1016/j.celrep.2024.113956 PMC11100346

[31] S. Morabito , F. Reese , N. Rahimzadeh , E. Miyoshi , and V. Swarup , “hdWGCNA Identifies Co‐Expression Networks in High‐Dimensional Transcriptomics Data,” Cell Reports Methods 3 (2023): 100498. 37426759 10.1016/j.crmeth.2023.100498 PMC10326379

[32] J. Yates , A. Kraft , and V. Boeva , “Filtering Cells With High Mitochondrial Content Depletes Viable Metabolically Altered Malignant Cell Populations in Cancer Single‐Cell Studies,” Genome Biology 26 (2025): 91. 40205439 10.1186/s13059-025-03559-w PMC11983838

[33] H. J. Kim , H. M. Jeon , D. C. Batara , et al., “CREB5 Promotes the Proliferation and Self‐Renewal Ability of Glioma Stem Cells,” Cell Death Discovery 10 (2024): 103. 38418476 10.1038/s41420-024-01873-z PMC10901809

[34] B. Tavora , T. Mederer , K. J. Wessel , et al., “Tumoural Activation of TLR3‐SLIT2 Axis in Endothelium Drives Metastasis,” Nature 586 (2020): 299–304. 32999457 10.1038/s41586-020-2774-y PMC8088828

[35] Y. Zhang , Y. Zhang , S. Kameishi , et al., “The Amot/Integrin Protein Complex Transmits Mechanical Forces Required for Vascular Expansion,” Cell Reports 36 (2021): 109616. 34433061 10.1016/j.celrep.2021.109616

[36] Y. Liu , X. Zhang , C. Han , et al., “TP53 Loss Creates Therapeutic Vulnerability in Colorectal Cancer,” Nature 520 (2015): 697–701. 25901683 10.1038/nature14418 PMC4417759

[37] Y. Zhang , M. Luo , X. Cui , D. O'Connell , and Y. Yang , “Long Noncoding RNA NEAT1 Promotes Ferroptosis by Modulating the miR‐362‐3p/MIOX Axis as a ceRNA,” Cell Death and Differentiation 29 (2022): 1850–1863. 35338333 10.1038/s41418-022-00970-9 PMC9433379

[38] W. S. Yang , R. SriRamaratnam , M. E. Welsch , et al., “Regulation of Ferroptotic Cancer Cell Death by GPX4,” Cell 156 (2014): 317–331. 24439385 10.1016/j.cell.2013.12.010 PMC4076414

[39] C. Mao , X. Liu , Y. Zhang , et al., “DHODH‐Mediated Ferroptosis Defence Is a Targetable Vulnerability in Cancer,” Nature 593 (2021): 586–590. 33981038 10.1038/s41586-021-03539-7 PMC8895686

[40] H. H. Jabara , S. E. Boyden , J. Chou , et al., “A Missense Mutation in TFRC, Encoding Transferrin Receptor 1, Causes Combined Immunodeficiency,” Nature Genetics 48 (2016): 74–78. 26642240 10.1038/ng.3465 PMC4696875

[41] S. Doll , B. Proneth , Y. Y. Tyurina , et al., “ACSL4 Dictates Ferroptosis Sensitivity by Shaping Cellular Lipid Composition,” Nature Chemical Biology 13 (2017): 91–98. 27842070 10.1038/nchembio.2239 PMC5610546

[42] X. Luan , X. Peng , G. Hui , and Z. Wei , “TFAP2A Promotes Cell Progression and Suppresses Ferroptosis in Lung Adenocarcinoma via Activating Transcription of CST1,” Journal of Biochemical and Molecular Toxicology 39 (2025): e70087. 39692484 10.1002/jbt.70087

[43] Z. Zhang , H. Xu , J. He , et al., “Inhibition of KLF5 Promotes Ferroptosis via the ZEB1/HMOX1 Axis to Enhance Sensitivity to Oxaliplatin in Cancer Cells,” Cell Death & Disease 16 (2025): 28. 39827156 10.1038/s41419-025-07330-8 PMC11743205

[44] Y. Wang , Q. Gao , X. Chen , et al., “TCF4 Promotes Neuroblastoma Proliferation and Inhibits Ferroptosis by Transactivating GPX4 Expression,” Applied Biochemistry and Biotechnology 197 (2025): 1–6268. 40668527 10.1007/s12010-025-05329-7

[45] H. Tang , R. Kang , J. Liu , and D. Tang , “ATF4 in Cellular Stress, Ferroptosis, and Cancer,” Archives of Toxicology 98 (2024): 1025–1041. 38383612 10.1007/s00204-024-03681-x

[46] N. Kuganesan , S. Dlamini , S. Hasan , L. V. Tillekeratne , and W. R. Taylor , “Regulation of Ferroptosis by Transcription Factor E2F1,” Biochimie 236 (2025): 127–137. 40578750 10.1016/j.biochi.2025.06.013 PMC12333391

[47] G. Lei , C. Mao , A. D. Horbath , et al., “BRCA1‐Mediated Dual Regulation of Ferroptosis Exposes a Vulnerability to GPX4 and PARP Co‐Inhibition in BRCA1‐Deficient Cancers,” Cancer Discovery 14 (2024): 1476–1495. 38552003 10.1158/2159-8290.CD-23-1220 PMC11296921

[48] H. Lee , A. Horbath , L. Kondiparthi , et al., “Cell Cycle Arrest Induces Lipid Droplet Formation and Confers Ferroptosis Resistance,” Nature Communications 15 (2024): 79. 10.1038/s41467-023-44412-7 PMC10761718 38167301

[49] R. Chen , L. Guo , Y. Chen , Y. Jiang , W. G. Wierda , and W. Plunkett , “Homoharringtonine Reduced MCL‐1 Expression and Induced Apoptosis in Chronic Lymphocytic Leukemia,” Blood 117 (2011): 156–164. 20971952 10.1182/blood-2010-01-262808 PMC3037741

[50] X. J. Chen , W. N. Zhang , B. Chen , et al., “Homoharringtonine Deregulates MYC Transcriptional Expression by Directly Binding NF‐kappaB Repressing Factor,” Proceedings of the National Academy of Sciences of the United States of America 116 (2019): 2220–2225. 30659143 10.1073/pnas.1818539116 PMC6369765

[51] K. Qin , S. Yu , Y. Liu , et al., “USP36 Stabilizes Nucleolar Snail1 to Promote Ribosome Biogenesis and Cancer Cell Survival Upon Ribotoxic Stress,” Nature Communications 14 (2023): 6473. 10.1038/s41467-023-42257-8 PMC10575996 37833415

[52] M. Ghandi , F. W. Huang , J. Jane‐Valbuena , et al., “Next‐Generation Characterization of the Cancer Cell Line Encyclopedia,” Nature 569 (2019): 503–508. 31068700 10.1038/s41586-019-1186-3 PMC6697103

[53] T. Naganuma , S. Nakagawa , A. Tanigawa , Y. F. Sasaki , N. Goshima , and T. Hirose , “Alternative 3′‐End Processing of Long Noncoding RNA Initiates Construction of Nuclear Paraspeckles,” EMBO Journal 31 (2012): 4020–4034. 22960638 10.1038/emboj.2012.251 PMC3474925

[54] H. Yan , Z. Wang , Y. Sun , L. Hu , and P. Bu , “Cytoplasmic NEAT1 Suppresses AML Stem Cell Self‐Renewal and Leukemogenesis Through Inactivation of Wnt Signaling,” Advanced Science (Weinheim, Germany) 8 (2021): e2100914. 10.1002/advs.202100914 PMC8596104 34609794

[55] Z. Zhang and J. Lieberman , “MALAT1 Protects Dormant Tumor Cells From Immune Elimination,” Nature Cancer 5 (2024): 218–220. 38195934 10.1038/s43018-023-00682-0

