# Supplementary figures and images for: Multiplexed Transcriptomics for Screening Drug Combinations and Defining the Mechanism of Action of HCC Therapeutics at Single‐Cell Resolution
Source: Cell Prolif. 2025 Nov 27;59(6):e70148. doi: 10.1111/cpr.70148 (PMC13241834; doi:10.1111/cpr.70148)

(A)

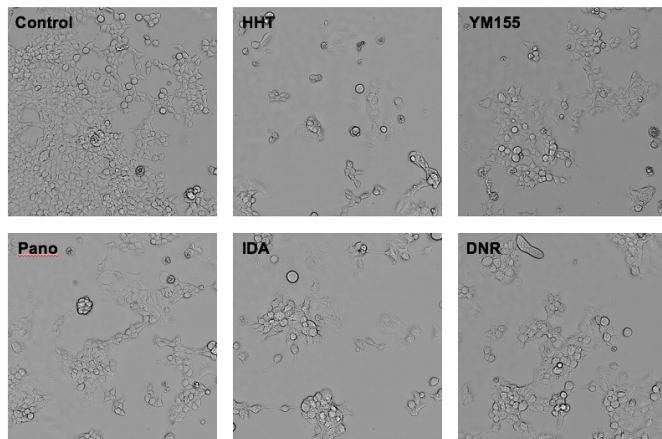

(B)

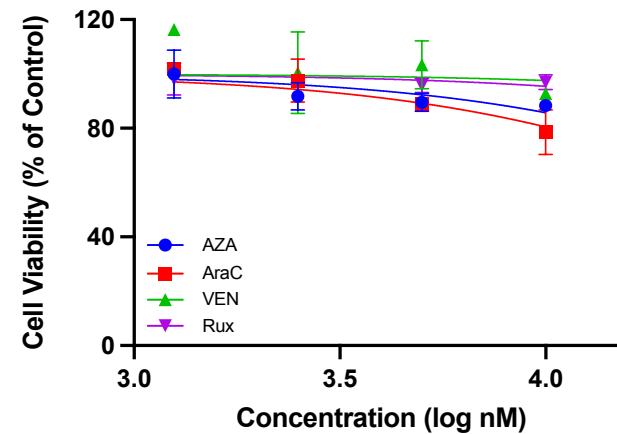

(C)

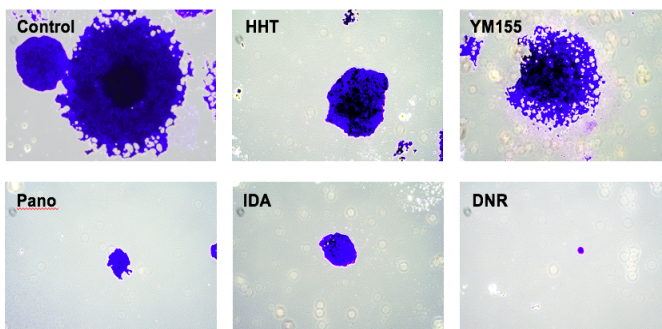

(D)

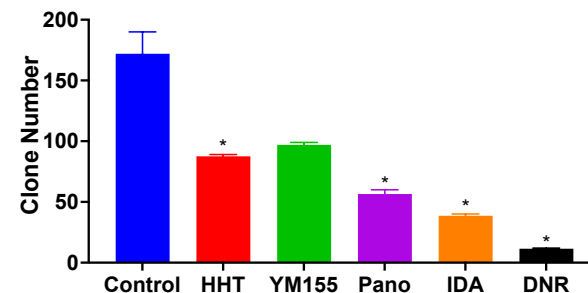

(A)

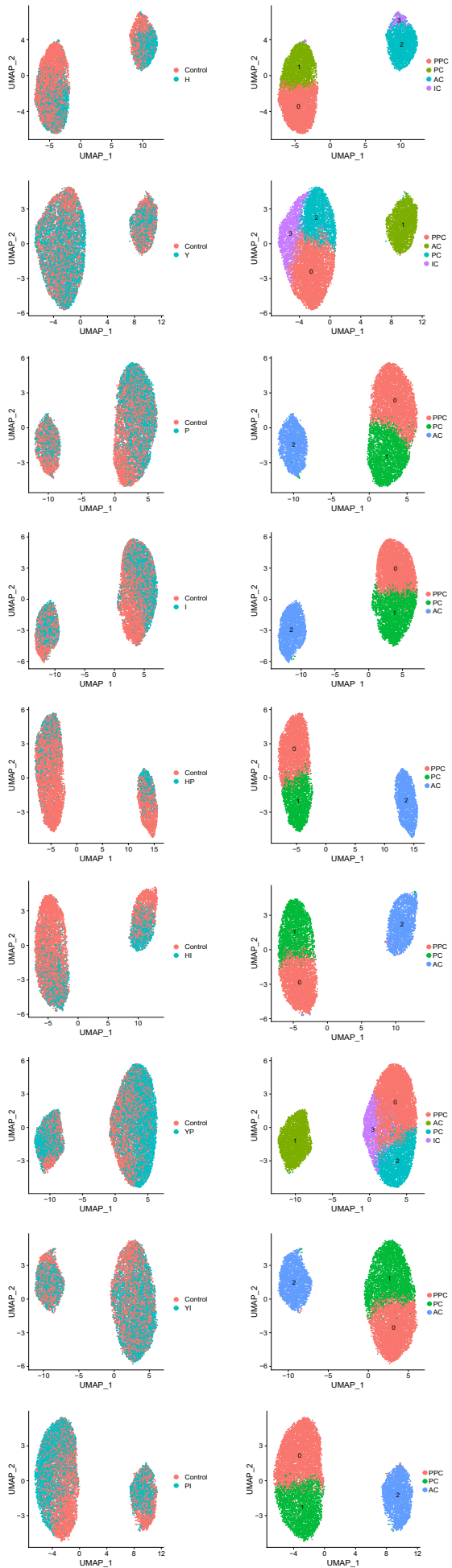

(B)

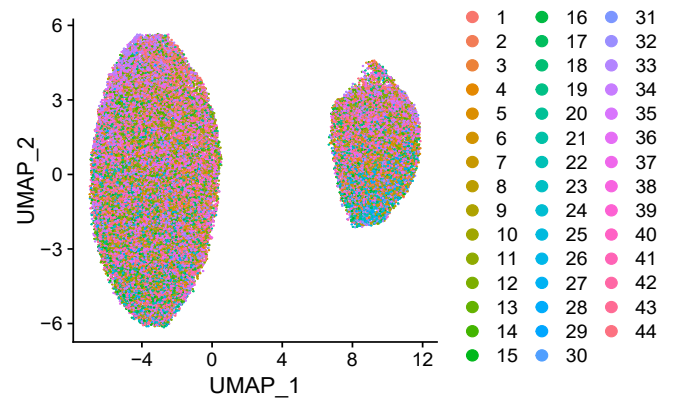

(C)

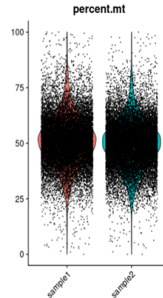

(D)

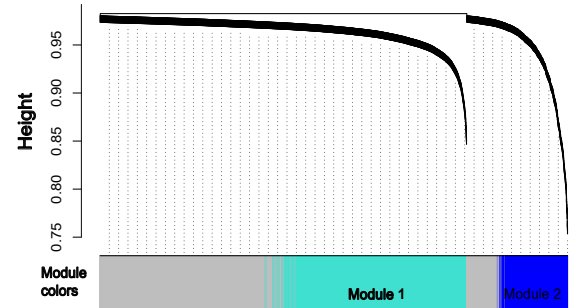

(E)

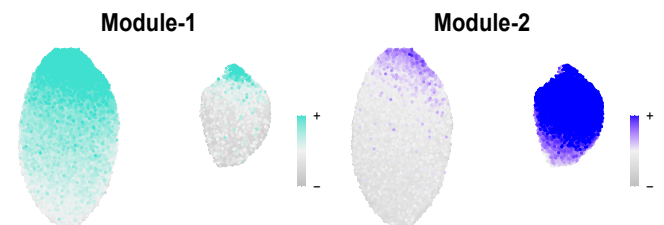

(F)

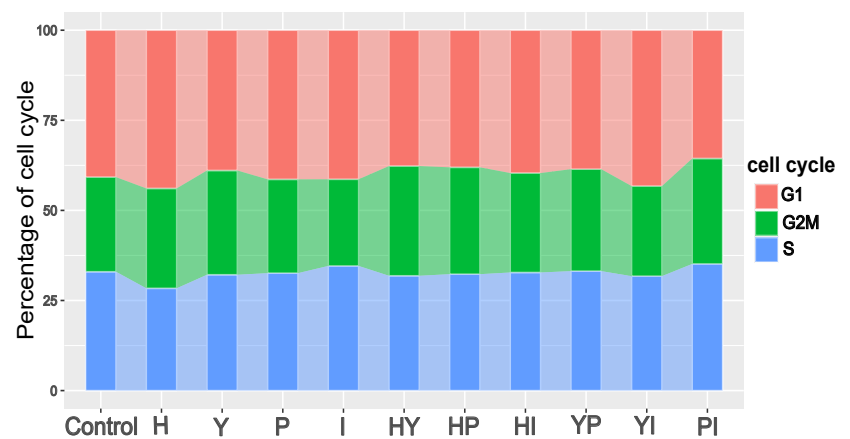

(A)

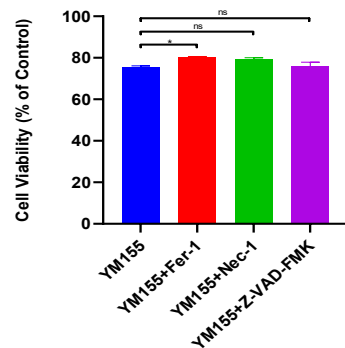

(B)

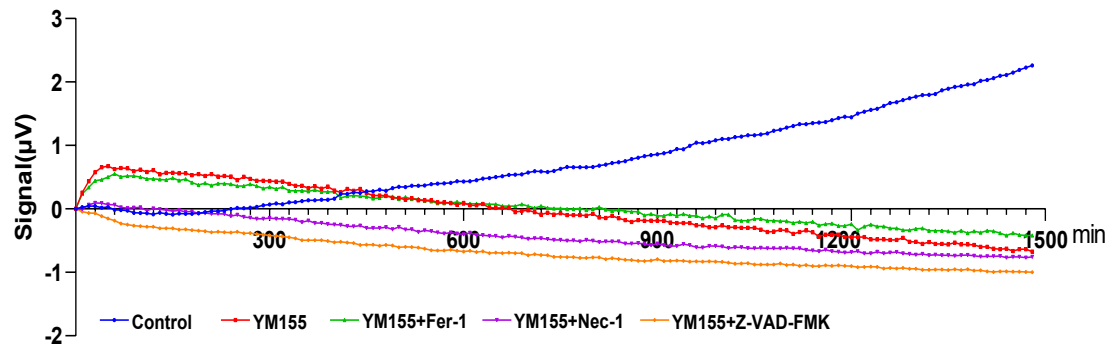

(C)

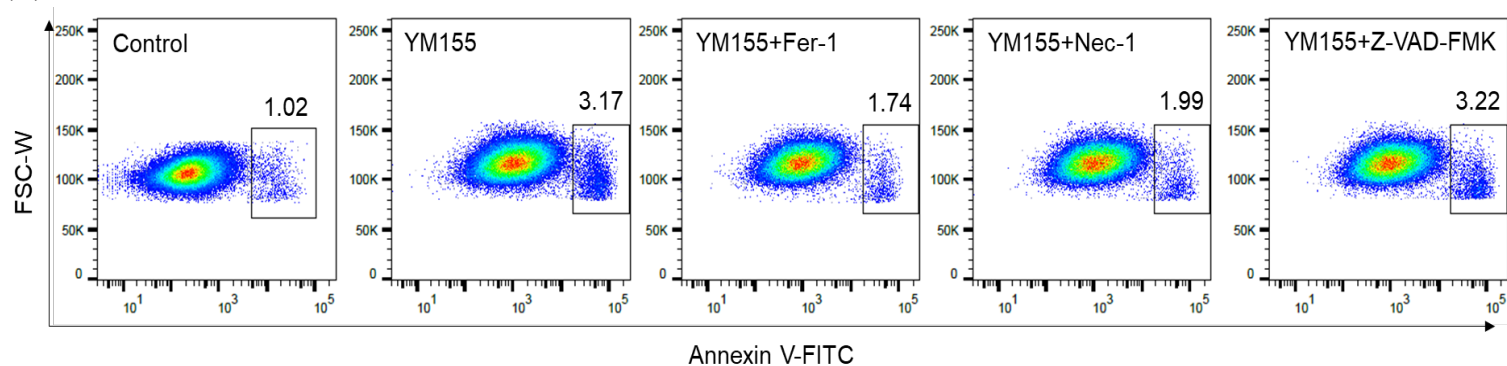

(D)

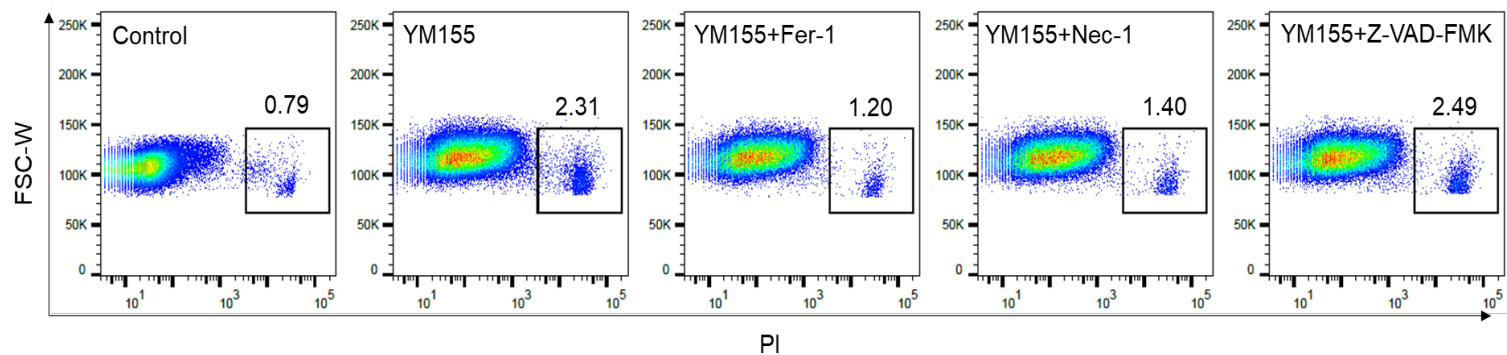

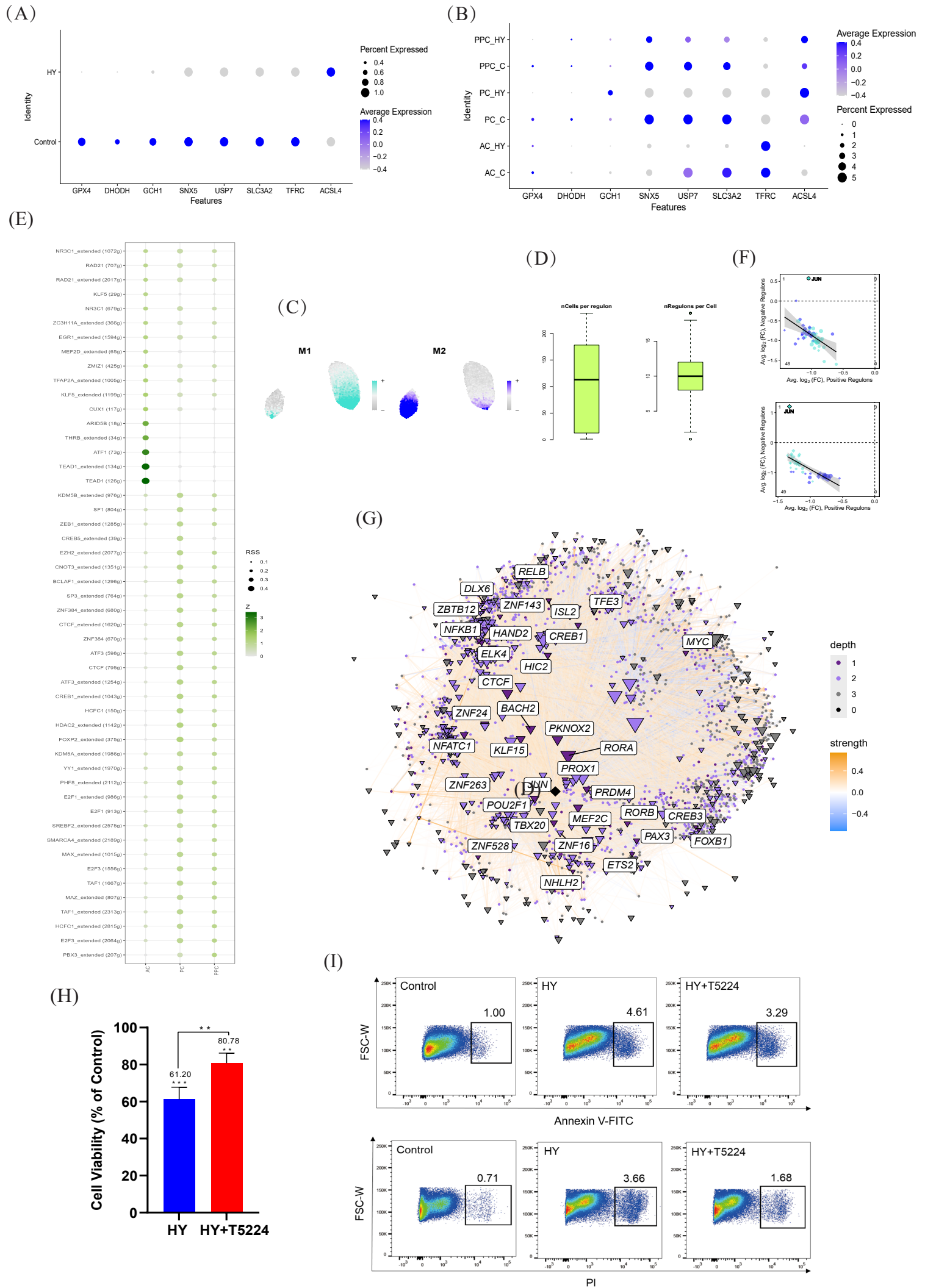

Supplement: Supplementary file 1 — Figure S1: Drug screening identified compounds that effectively inhibit HCC cell proliferation. (A) Images of HepG2 cell morphology. (B) Cell viability percentage (%) of AZA, AraC, VEN, and Rux against HepG2 cell line. Cells were seeded in a 96‐well plate and treated with 1.25 μM, 2.5 μM, 5 μM, 10 μM drug for 48 h, and the cell viability percentage was detected with CCK‐8 kit. (C) Colony formation assay was conducted to investigate tumour growth after treatment with HHT, YM155, Pano, IDA, and DNR for 14 days. The colonies were visualised with the images. (D) The corresponding histogram showed the colony numbers. Data were presented as mean ± SD (n = 3) and comparisons were performed with unpaired two‐tailed Student's t test. *p < 0.05. The absence of a * mark indicates no statistical significance. Figure S2: Single‐cell screening uncovered the heterogeneous transcriptional response characteristics. (A) UMAP embedding of the HCC cells analysed in this study. Colour‐coded for specific drug treatment (left) and cell type (right). (B) UMAP embedding of the HCC cells analysed in this study. Colour‐coded for RT barcode. (C) The violin plot of chrMT% distribution. (D) HdWGCNA analysis of HCC cells with different drug treatment identified two modules. (E) UMAP embedding of the HCC cells analysed in this study. Colour‐coded for module 1 (left) and module 2 (right). (F) Bar chart showing the percentage of cell cycle in HCC cells with different drug treatment. H, HHT; Y, YM155; P, Pano; I, IDA; HY, HHT&YM155; HP, HHT&Pano; HI, HHT&IDA; YP, YM155&Pano; YI, YM155&IDA; PI, Pano&IDA; PPC, primary proliferative cells; PC, proliferation‐related cells; AC, apoptosis‐related cells; IC, intermediate cells. Figure S3: Functional experiments validated the inhibitory efficiency of drug combination. (A) Cell viability percentage (%) of YM155, YM155&Fer‐1, YM155&Nec‐1, and YM155&Z‐VAD‐FMK against HepG2 cell line. Cells were seeded in 96‐well plate for 48 h, and the cell viability percentage [file CPR-59-e70148-s004.pdf]
