# Supplementary material for: Multiplexed Transcriptomics for Screening Drug Combinations and Defining the Mechanism of Action of HCC Therapeutics at Single‐Cell Resolution
Source: Cell Prolif. 2025 Nov 27;59(6):e70148. doi: 10.1111/cpr.70148 (PMC13241834; doi:10.1111/cpr.70148)
Supplement: Supplementary file 5 — Data S1: Supporting Information. [file CPR-59-e70148-s001.docx]

**Supplementary Fig.1 Drug screening identified compounds that effectively inhibit HCC cell proliferation. (A)** Images of HepG2 cell morphology. **(B)** Cell viability percentage (%) of AZA, AraC, VEN, and Rux against HepG2 cell line. Cells were seeded in a 96-well plate and treated with 1.25μM, 2.5μM, 5μM, 10μM drug for 48h, and the cell viability percentage was detected with CCK-8 kit. **(C)** Colony formation assay was conducted to investigate tumor growth after treatment with HHT, YM155, Pano, IDA, and DNR for 14 days. The colonies were visualized with the images. **(D)** The corresponding histogram showed the colony numbers. Data were presented as mean±SD (n=3) and comparisons were performed with unpaired two-tailed Student’s t test. *p<0.05. The absence of a * mark indicates no statistical significance.

**Supplementary Fig.2 Single-cell screening uncovered the heterogeneous transcriptional response characteristics. (A)** UMAP embedding of the HCC cells analyzed in this study. Color-coded for specific drug treatment (left) and cell type (right). **(B)** UMAP embedding of the HCC cells analyzed in this study. Color-coded for RT barcode. **(C)** The violin plot of chrMT% distribution. **(D)** HdWGCNA analysis of HCC cells with different drug treatment identified two modules. **(E)** UMAP embedding of the HCC cells analyzed in this study. Color-coded for module 1 (left) and module 2 (right). **(F)** Bar chart showing the percentage of cell cycle in HCC cells with different drug treatment. H, HHT; Y, YM155; P, Pano; I, IDA; HY, HHT&YM155; HP, HHT&Pano; HI, HHT&IDA; YP, YM155&Pano; YI, YM155&IDA; PI, Pano&IDA; PPC, primary proliferative cells; PC, proliferation-related cells; AC, apoptosis-related cells; IC, intermediate cells.

**Supplementary Fig.3 Functional experiments validated the inhibitory efficiency of drug combination. (A)** Cell viability percentage (%) of YM155, YM155&Fer-1, YM155&Nec-1, and YM155&Z-VAD-FMK against HepG2 cell line. Cells were seeded in 96-well plate for 48h, and the cell viability percentage was detected with CCK-8 kit. **(B)** Real-time signal analysis of cell proliferation after treatment with YM155, YM155&Fer-1, YM155&Nec-1, and YM155&Z-VAD-FMK. **(C)** Early apoptosis percentage (top) and late apoptosis percentage (bottom) of YM155, YM155&Fer-1, YM155&Nec-1, and YM155&Z-VAD-FMK against HepG2 cell line. Cells were seeded with different drug combination in 6-well plate for 48h, and the Annexin V-FITC and PI expression was detected by LSR Fortessa flow cytometry. Data were presented as mean±SD (n=3) and comparisons were performed with unpaired two-tailed Student’s t test. *p<0.05, ns, no significance.

**Supplementary Fig.4 Molecular mechanisms elucidation at the single-cell level. (A)** Dot plot showing the expression of signature genes associated with ferroptosis in HCC with (HY) or without (Control) HY treatment. Average expression represents the average expression level of a specific gene within a defined cell population. Percent expressed refers to the proportion of cells within the given population that show detectable expression of the gene. **(B)** Dot plot showing the expression of signature genes associated with ferroptosis across different cell types with (HY) or without (C) HY treatment. Average expression represents the average expression level of a specific gene within a defined cell population. Percent expressed refers to the proportion of cells within the given population that show detectable expression of the gene. **(C)**UMAP embedding of the HCC cells with HY treatment. Color-coded for module 1 (left) and module 2 (right). **(D)** Bar chart showing the number of cells per regulon (left) and the number of regulons per cell (right). **(E)** Dot plot showing the representative regulon in each cluster of HCC cells with HHT treatment. Regulon specificity score (RSS) measures the specificity score of a regulon across different cell types. Z score assesses the expression level of an individual gene relative to its background distribution, measured in standard deviations. **(F)** Scatter plot showing the effect sizes from the differential regulon test for the positive (x-axis) and negative (y-axis) regulons. For the TFs in the top left corner, the negatively-correlated target genes are up-regulated of AC cluster in HY treatment relative to control (left), and the negatively-correlated target genes are up-regulated of in HY treatment relative to control (right). Each point represents a TF, colored by the module assignment in **(C)**. Diamonds represent TFs that are also significantly differentially expressed, while circles are not differentially expressed. TFs that did not reach significance are opaque while the significant TFs have a black outline. The number of significantly differentially expressed regulons in each quadrant of the plot are labeled in the corners. **(G)** The TF network showing regulatory links originating from JUN. The nodes represent TFs and genes, and the edges represent inferred regulatory relationships. The selected TF is shown as a diamond, other TFs are shown as triangles, and genes are shown as circles. The size of each node corresponds to the outdegree in the network. The color of the edges represents the strength of the TF-gene interaction based on the pearson correlation of gene expression. The color of each node represents the number of links to the selected TFs. **(H)** Cell viability percentage (%) of HY and HY+T5224 against HepG2 cell line. Cells were seeded in a 96-well plate for 48h, and the cell viability percentage was detected with CCK-8 kit. **(I)** Early apoptosis percentage (upper) and late apoptosis percentage (below) of HY and HY+T5224 against HepG2 cell line. Cells were seeded with different drug combination in 6-well plate for 48h, and the Annexin V-FITC and PI expression was detected by LSR Fortessa flow cytometry. HY, HHT&YM155; PPC, primary proliferative cells; PC, proliferation-related cells; AC, apoptosis-related cells. Data were presented as mean±SD (n=3) and comparisons were performed with unpaired two-tailed Student’s t-test. *p<0.05, **p<0.01, ***p<0.001.

**Supplementary Table 1 Drug screening identified compounds that effectively inhibit HCC cell proliferation.** The information of drugs used in this study, including full name, abbreviation, specification, solvent and aliquot concentration.

**Supplementary Table 2 Single-cell screening uncovered the heterogeneous transcriptional response characteristics. sheet 1** The RT barcode information corresponding to each drug treatment group. **sheet 2** The cell number of each cell type information corresponding to each drug treatment group. **sheet 3** The cell number of each cell cycle information corresponding to each drug treatment group. **sheet 4-14** Differentially expressed genes detected in each cell type for HCC cells with different drug treatment. Yellow labels indicate specific marker genes of cell clusters. Genes are selected by log foldchange > 0.25, Bonferroni-adjusted p-value < 0.1, expressed in at least 15% of cells in either population (Seurat FindAllMarkers). Log fold change is calculated as arithmetic mean of log10 cpm values of one population minus the arithmetic mean of log10 cpm values of the second, and fold change is 10log_foldchange. P-values were calculated by the Wilcoxon rank sum test.

**Supplementary Table 3 Molecular mechanisms elucidation at the single-cell level. sheet 1-3** Differentially expressed genes detected in each cell cluster for HCC cells with HY treatment. Genes are selected by log foldchange > 0.25, Bonferroni-adjusted p-value < 0.1, expressed in at least 15% of cells in either population (Seurat FindAllMarkers). Log fold change is calculated as arithmetic mean of log10 cpm values of one population minus the arithmetic mean of log10 cpm values of the second, and fold change is 10log_foldchange. P-values were calculated by the Wilcoxon rank sum test. **sheet 4** The information of hub genes in HCC cells with HY treatment. **sheet 5** The information of enriched motif in HCC cells with HY treatment. **sheet 6** The information of regulon and target genes in HCC cells with HY treatment. **sheet 7** The information of AUC cell thresholds in HCC cells with HY treatment.
